# Behavioural and psychological symptoms of people with dementia in acute hospital settings: a systematic review and meta-analysis

**DOI:** 10.1093/ageing/afaf013

**Published:** 2025-01-31

**Authors:** Kanthee Anantapong, Aimorn Jiraphan, Warut Aunjitsakul, Katti Sathaporn, Nisan Werachattawan, Teerapat Teetharatkul, Pakawat Wiwattanaworaset, Nathan Davies, Elizabeth L Sampson

**Affiliations:** Department of Psychiatry, Faculty of Medicine, Prince of Songkla University, Hat Yai, Songkhla, 90110, Thailand; Department of Psychiatry, Faculty of Medicine, Prince of Songkla University, Hat Yai, Songkhla, 90110, Thailand; Department of Psychiatry, Faculty of Medicine, Prince of Songkla University, Hat Yai, Songkhla, 90110, Thailand; Department of Psychiatry, Faculty of Medicine, Prince of Songkla University, Hat Yai, Songkhla, 90110, Thailand; Department of Psychiatry, Faculty of Medicine, Prince of Songkla University, Hat Yai, Songkhla, 90110, Thailand; Department of Psychiatry, Faculty of Medicine, Prince of Songkla University, Hat Yai, Songkhla, 90110, Thailand; Department of Psychiatry, Faculty of Medicine, Prince of Songkla University, Hat Yai, Songkhla, 90110, Thailand; Centre for Psychiatry and Mental Health, Wolfson Institute of Population Health, Queen Mary University of London, London, E13 8SP, United Kingdom of Great Britain and Northern Ireland; Centre for Psychiatry and Mental Health, Wolfson Institute of Population Health, Queen Mary University of London, London, E13 8SP, United Kingdom of Great Britain and Northern Ireland; Academic Centre for Healthy Ageing, Whipps Cross Hospital, Barts Health NHS Trust, Queen Mary University of London, London, E11 1NR, United Kingdom of Great Britain and Northern Ireland; Department of Psychological Medicine, Royal London Hospital, East London NHS Foundation Trust, London, E1 1BJ, United Kingdom of Great Britain and Northern Ireland

**Keywords:** dementia, cognitive impairment, psychiatric symptom, hospital care, inpatient, older people, systematic review

## Abstract

**Background:**

Behavioural and psychological symptoms of dementia (BPSD) can complicate acute hospital care, but evidence on BPSD in this setting is heterogeneous.

**Objective:**

To determine the prevalence of BPSD in acute hospitals and explore related risk factors, treatments, and outcomes (PROSPERO: CRD42023406294).

**Methods:**

We conducted a systematic review and meta-analysis by searching Cochrane Library, MEDLINE, and PsycINFO for studies on BPSD prevalence among older people with dementia during their acute hospital admissions (up to 5 March 2024). Independent double-review processes were used for study screening, selection, and data extraction. Data on 12 BPSD symptoms were extracted based on the Neuropsychiatric Inventory Questionnaire (NPI) and Behavioural Pathology in Alzheimer’s Disease (BEHAVE-ad). Risk factors, treatments, and outcomes were summarised. Meta-analysis was used to synthesise results.

**Results:**

Out of 15 101 records, 30 articles from 23 studies were included. Most studies were rated as moderate (n = 12) to poor (n = 17) quality. Meta-analysis revealed a pooled prevalence of overall BPSD (one or more BPSD symptoms) at 60% (95% CI = 43–78%) among older inpatients with dementia in acute hospitals (N = 11 studies). Subgroup analysis showed variations in the overall BPSD prevalence based on assessment tools (BEHAVE-ad = 85%, NPI = 74%, Others = 40%). Common BPSD symptoms included aggression/agitation (39%), sleep problems (38%), eating problems (36%), and irritability (32%). BPSD were linked to delirium, pain, increased use of uncomfortable interventions, psychotropic medication uses and higher caregiver distress. Poor patient-staff interactions and fragmented discharge plans often led to frequent emergency admissions and hospital readmissions.

**Conclusion:**

Healthcare systems should implement tailored approaches for managing BPSD in acute hospitals, enhance staff training, improve caregiver communication, and develop integrated discharge plans.

## Key Points

- Acute hospital admissions of people with dementia can worsen behavioural and psychological symptoms (BPSD) and further complicate care.
- Sixty percent of older hospital patients with dementia have one or more BPSD symptoms, including aggression, sleep issues, eating problems, and irritability.
- BPSD are linked to delirium, pain, uncomfortable interventions, psychotropic use, caregiver distress, poor discharge planning, and frequent readmissions.
- Using standard assessment tools can help identify and manage BPSD in hospitalised older patients with dementia.

## Introduction

During the course of dementia, up to 90% of individuals are likely to experience some form of behavioural and psychological symptoms of dementia (BPSD). These symptoms often contribute to poor care outcomes, such as prolonged hospitalisation, inappropriate medication use, and increased health and social care costs [1]. BPSD encompasses a range of symptoms, including depression, anxiety, apathy, elation, irritability, aggression, psychosis (hallucinations and delusions), abnormal motor behaviours, and eating problems [2].

Family carers and healthcare professionals often find these symptoms distressing, as does the person with dementia and BPSD, which are challenging to manage [3–6]. Nonpharmacological interventions, such as improving the social and physical environment, providing caregiver training and education, and offering cognitive rehabilitation, are recommended as first-line approaches before considering pharmacological treatments. This preference is due to the potential for drug interactions, side effects, and increased risks of morbidity and mortality associated with some pharmacological options [7]. However, implementing non-pharmacological interventions can be resource-intensive, requiring significant time and staffing, and most research on these interventions has been conducted in long-term care settings, making it challenging to apply them in other contexts [8, 9].

Managing BPSD in acute hospital settings is particularly challenging due to the busy environment, limited staffing, and rigid care routines, which often prevent systematic implementation and monitoring of BPSD management [10, 11]. Acute conditions, particularly delirium can trigger, worsen, and overlap with BPSD symptoms [12, 13]. Additionally, healthcare professionals may have varying levels of expertise in dementia care, especially in managing BPSD [5, 14]. As a result, people with dementia are at a higher risk of developing BPSD and experiencing its negative consequences when hospitalised; however, BPSD are likely to go unrecognised in acute hospitals [15]. Assessing BPSD in acutely ill patients with dementia is challenging due to environmental and physiological changes affecting cognition and may not accurately reflect prehospitalisation symptoms. But, such assessments in acute hospital settings are valuable for early recognition, proper management, and careful follow-up of these symptoms.

Several studies have highlighted the prevalence and characteristics of BPSD in hospital settings. For example, Sampson et al. [16] reported that 75% of people with dementia admitted for acute medical illnesses had BPSD, with common symptoms including aggression (57%), activity disturbance (44%), sleep disturbance (42%), and anxiety (35%). Of these, 43% had symptoms that were moderately to severely distressing for staff. Similarly, Djekovic et al. [17] found that 49% of patients with BPSD in a specialist dementia care unit had symptoms classified as ‘high severity’.

Despite these findings, there has been no systematic review specifically focusing on the prevalence of various BPSD in people with dementia within acute hospital settings. Existing reviews have primarily addressed community or mixed settings (e.g. Kwon and Lee [18], Zhao et al. [19] and Selbaek et al. [20]. Therefore, this systematic review aims to determine the prevalence of BPSD in acute hospitals, with secondary objectives to examine risk factors and outcomes, including psychotropic use, falls, length of stay (LOS), rehospitalization, long-term care placement, caregiver distress, mortality, and care costs.

## Methods

### Design

A systematic review of quantitative studies was conducted using a meta-analysis. We followed the Preferred Reporting Items for Systematic Reviews and Meta-Analysis (PRISMA) Statement in reporting the review [21] (see Appendix 1 in the Supplementary Data section for the full details). The review protocol was registered on PROSPERO (CRD42023406294).

### Criteria for inclusion

We selected peer-reviewed original research articles that reported on the prevalence of BPSD in acute hospital settings, adhering to the criteria outlined in Table 1, guided by previous studies [18, 22, 23]. There was no limitation regarding the year of publication.

**Table 1:** Inclusion and exclusion criteria for eligible studies.

**Inclusion criteria**
Participant: People with dementia (any subtypes and stages) who admitted to an acute hospital previously diagnosed with dementia OR newly diagnosed with dementia in the study
Study design: Original quantitative study – observational design
Setting: Acute general hospital (including a psychiatric unit of general hospitals)
Outcome: A study reported prevalence of BPSD (either individual OR overall BPSD prevalence), determined by standardised assessment tools or clinical judgement/criteria by clinical team an individual BPSD/neuropsychiatric symptom (informed by Neuropsychiatric Inventory Questionnaire (NPI-Q) and Behavioural Pathology in Alzheimer’s Disease (BEHAVE-ad)): depression, anxiety, apathy, elation/mania, agitation/aggression, delusion, hallucination, wandering/aberrant motor, sleep problems, eating problems (the included studies may explore only one or more of these symptoms) overall BPSD = any BPSD above (people who have one or more individual BPSD symptom)
**Exclusion criteria**
Participant: people with cognitive impairment but not formally diagnosed with dementia older people with dementia with other serious illness that can mimic BPSD, including cancer, Down’s syndrome, delirium (in the absence of dementia), schizophrenia, bipolar disorder, major depressive disorder, post-traumatic stress disorder
Study design: Controlled interventional study, qualitative study, case study, review, editorial, conference proceeding, thesis
Setting: Nursing or residential home, community, psychiatric hospital, dementia specialist unit, long-term or rehabilitation hospital, outpatient including memory clinic, respite care
Others: Studies not published in English and unpublished manuscripts were excluded due to resource constraints.

Due to expected variations of diagnostic criteria of dementia, we included studies that either used existing medical records giving a clinical dementia diagnosis, or based on Diagnostic and Statistical Manual of Mental Disorders (DSM) and International Classification of Diseases (ICD) or clinical assessment tools in the studies, e.g. the Informant Questionnaire on Cognitive Decline in the Elderly (IQCODE), and the Mini Mental State Examination (MMSE). To cover a range of acute care settings, we included those that commonly treat acute physical health conditions of people with dementia, such as acute geriatric wards, medical wards, psychogeriatric wards, surgical wards, and intensive care units.

### Search strategy

The following electronic databases were searched for peer-reviewed articles: Cochrane Library, MEDLINE (via Ovid), and PsycINFO (via Ovid). To perform the search, a combination of Medical Subject Headings (MeSH), subject headings, and keywords was developed using Boolean operators, with appropriate synonyms and abbreviations added. The search was initially informed by previous systematic reviews on BPSD [18, 24], the Neuropsychiatric Inventory Questionnaire (NPI-Q) [22] and Behavioural Pathology in Alzheimer’s Disease (BEHAVE-ad) rating scale [23]. An initial literature scoping helped refine the search terms, which were customised for each database (see Appendix 2 for the full search strategy). We collaborated with information specialists at the Faculty of Medicine Library, Prince of Songkla University, to ensure the search strategy was accurate and comprehensive. Citation tracking and reference list checks of identified eligible articles were conducted to find additional relevant articles. We also consulted with experts in the field to identify any missed articles. The database search was conducted up to 5 March 2024.

### Selection procedure

Following the established inclusion criteria, citations were independently screened and evaluated against the inclusion/exclusion criteria, initially based on article titles and abstracts, by at least two reviewers (KA or AJ, along with one of WA, KS, TT, or PW). Any disagreements were resolved through discussion with a third reviewer to reach consensus. The full texts of the selected titles and abstracts were then independently reviewed and assessed against the inclusion criteria by two reviewers (KA or AJ, and one of WA, KS, TT, or PW). The study selection went to a third reviewer (ES or ND) for discussion.

### Data extraction

Relevant data from the included studies were extracted and recorded using a standardised data extraction form developed in Microsoft Excel. The data items sought and extracted were listed in the full data extraction form (see Appendix 3). The form was piloted on six papers by three reviewers (KA, AJ, and WA), followed by a review meeting with ND and ELS to assess its comprehensiveness and practicality. Discrepancies were resolved through discussion with ND and ELS. Data extraction for the remaining papers was carried out by two reviewers (KA or AJ, and one of WA, KS, TT, or PW) using the finalised data extraction form. Attempts were made to contact study authors if information was missing.

### Quality assessment

We used the Joanna Briggs Institute (JBI) Critical Appraisal Checklist for Studies Reporting Prevalence Data to assess the quality of eligible studies [25]. One reviewer (KA, AJ, WA, KS, TT, or PW) conducted the quality assessment independently, while a second author (KA, ND, or ELS) verified the judgements. Any disagreements were resolved through discussion.

### Strategy for data synthesis

As part of the synthesis process, KA and NW tabulated the results and developed an initial synthesis framework, which was then reviewed and refined in a meeting with ND and ELS. KA used this agreed-upon framework to synthesise the included papers, with ongoing team discussions. KA and NW conducted statistical analyses, including meta-analysis, heterogeneity tests, and sensitivity analyses.

We reported the prevalence of overall BPSD and specific BPSD symptoms from the included studies using narrative, graphical, or tabular summaries. A meta-analysis using random effects was performed to pool the prevalence rates of overall BPSD and individual symptoms in acute hospital settings. To estimate the pooled prevalence of overall BPSD, we included only studies that examined two or more BPSD symptoms and reported the prevalence of overall BPSD (one or more BPSD symptoms) in the meta-analysis. Risk factors and outcomes related to overall BPSD and individual symptoms were summarised narratively and in tables. To explore sources of heterogeneity, we planned subgroup analyses based on dementia stage and subtype, hospital ward settings (surgical vs. non-surgical, ICU vs. non-ICU), and assessment tools. However, conducting a meta-analysis of prevalence studies can be challenging due to the high heterogeneity expected from the observational designs of the included studies [26, 27]. To address and understand this heterogeneity, we calculated relevant statistics, such as I^2^ and sensitivity analyses [27, 28].

## Results

### Study characteristics

**Figure 1:**
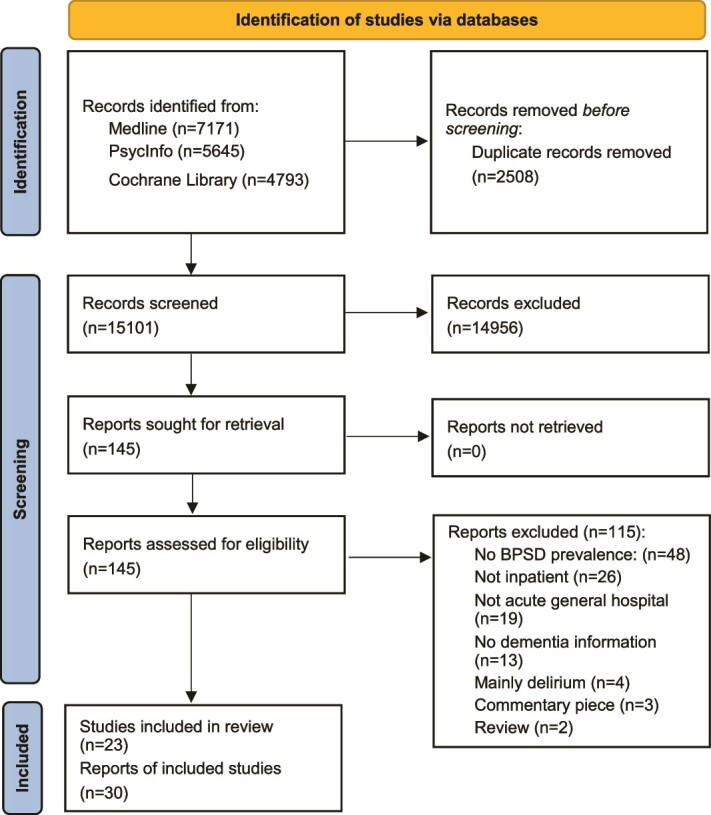
presents the PRISMA flowchart.

In this review, we included 30 published articles from 23 studies (see Figure 1 for PRISMA flowchart). The majority of articles came from Western countries, with nine from the USA [29–37], seven from the UK [16, 38–43], and others (7 articles) from Europe [5, 37, 44–48]. There were fewer articles (6 articles) from Asian countries [49–54], and one article from Australia [55]. The studies involved 109 805 older patients (range 42 to 53 156 patients) admitted to acute hospitals. Most articles reported a female predominance and a mean participant age over 70 years. The stage of dementia and admitting conditions were inconsistently reported. Each study examined 1–12 BPSD symptoms using various assessment tools, predominantly the NPI and BEHAVE-ad. Appendix 4 provides a summary of the characteristics of the included articles.

Only four included papers from a single study explicitly excluded people with delirium from their study, using the Confusion Assessment Method (CAM) [10, 16, 39, 40], while 14 papers did not perform or report delirium assessments (see Appendix 4 for more details). Twelve papers reported that they retrieved delirium diagnoses from the medical records [35, 36, 38, 45, 55, 56] or used standard delirium assessments to determine delirium [5, 29–31, 37, 48]. Some argued that the comorbidity reflects the clinical reality, so they decided not to exclude cases with delirium [5, 56], while others also used delirium as a covariate in their statistical analysis.

### Quality assessment

Most of the included studies were rated as moderate (n = 11) to good (n = 13) quality (see Appendix 5). Studies rated as poor or moderate often exhibited misclassification bias and potential confounding effects. Studies relying on retrospective chart reviews were at risk of underdiagnosing dementia and BPSD due to inconsistent recording of diagnoses and symptoms in routine practice. Additionally, delirium was not formally assessed in most studies, which may have affected the reported prevalence of BPSD in acute hospital settings.

### Main findings

#### Prevalence of overall and individual BPSD

Eleven studies were included to estimate the pooled prevalence of the overall BPSD symptoms in older adults in acute hospital settings.

**Figure 2:**
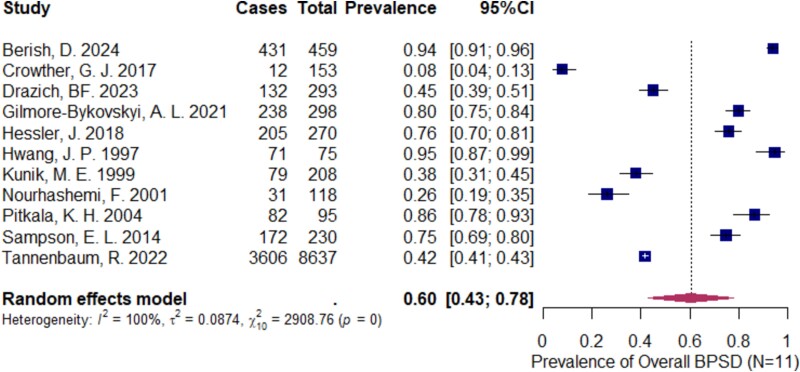
Forest plot shows the prevalence reported in the included studies for overall BPSD symptoms in acute hospital setting (N = 11)

From Figure 2, we analysed the studies reporting the prevalence of any BPSD symptoms in older adults in acute hospital settings and estimated the pooled prevalence of overall BPSD symptoms. The prevalence of overall BPSD across the 11 studies ranged from 8% to 95%. The pooled prevalence of overall BPSD was 60% (95% CI: 43–78%), with high heterogeneity (I^2^ = 100%, *P* = 0).

**Figure 3:**
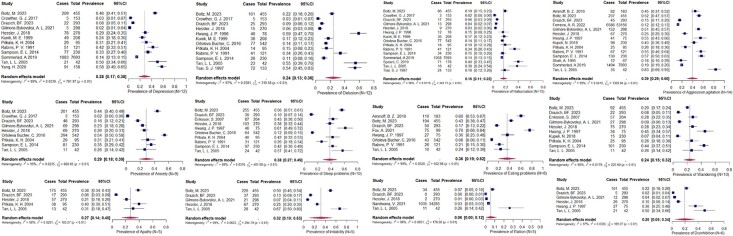
Forest plots show the prevalence reported in the included studies for individual BPSD symptoms in the acute hospital

Figure 3 shows the pooled prevalence of individual BPSD symptoms in older adults with dementia in acute hospitals. Aggression/agitation (39%), sleeping problems (38%), eating problems (36%), and irritability (32%) were most prevalent. Mood symptoms like anxiety (29%), depression (28%), and apathy (27%) were common. Psychosis, including hallucination (16%) and delusion (24%), affected about one in five patients. Elation was least common (6%). Depression, delusion, hallucination, and aggression/agitation were most frequently studied, while apathy, irritability, elation, and disinhibition were less examined. Significant heterogeneity was observed across analyses.

#### Subgroup analysis and sensitivity analysis

We conducted subgroup analyses based on assessment tools for BPSD (NPI, BEHAVE-ad, and other methods, including diagnostic interviews). Due to insufficient and inconsistent data in the included studies, we were unable to perform subgroup analyses based on ward setting, dementia subtype, and dementia severity as originally planned.

**Figure 4:**
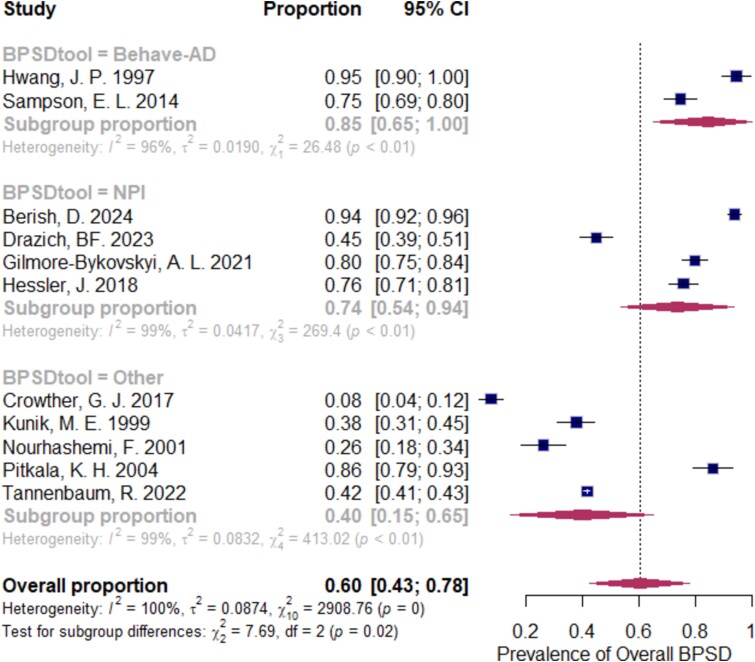
Forest plot shows subgroup analysis for overall BPSD prevalence based on assessment tools

Figure 4 shows a significantly higher prevalence of overall BPSD when using standard assessment tools, with a slightly higher prevalence reported with BEHAVE-ad (85%, 95% CI: 65–100%) compared to NPI (74%, 95% CI: 54–94%). Other assessment methods, including diagnostic interviews and chart reviews, yielded a lower prevalence (40%, 95% CI: 15–65%) (χ^2^ = 14.87, df = 2, *P* < .01).

A sensitivity analysis for delirium screening was conducted by removing one study with delirium exclusion criteria [16]. The results remained stable, with only slight changes in the overall proportion (60% to 59%) and confidence intervals (43–78% to 40–78%). The τ^2^ value showed a small increase, indicating consistent findings even with the study’s exclusion. (see Appendix 6).

#### Risk factors of BPSD

In this study, we summarised the included studies that reported on risk factors for BPSD in acute hospitals (see Appendix 7 for a detailed summary). Inpatients with BPSD were more likely to be male and White [56] and to be transferred from residential care facilities [16, 56]. Additionally, older inpatients were more prone to developing agitation during urgent admissions [45].

Severity of cognitive impairment was identified as a risk factor for developing BPSD during acute hospital admissions in several studies [5, 35, 51]. BPSD and delirium frequently co-existed in acute settings [16, 30, 37]. Pain was found to either cause or exacerbate BPSD, particularly agitation and aggression [31, 39, 40]. Additionally, poor quality of interactions between hospital professionals and patients with dementia was associated with BPSD [31]. (see Appendix 7 for more details).

#### Treatment and outcomes of BPSD

We synthesised the included studies that reported on the treatment and outcomes of BPSD in acute hospitals (see Appendix 8 for a detailed summary). During hospital admissions, individuals with BPSD were more likely to receive aggressive or uncomfortable interventions, such as bladder catheters, bed alarms, invasive mechanical ventilation, and restraints [48, 56]. Older inpatients with BPSD were more likely to require specialty consultations with palliative care, psychiatry, and neurology [5, 36, 56]. Only about half of patients with dementia received non-pharmacological treatments, with limited evidence on the effectiveness of these interventions [10]. A significant proportion of inpatients with dementia were prescribed psychotropic medications for BPSD, including antipsychotics, anxiolytics, hypnotics, and antidepressants [36, 56]. However, these medications carried risks of adverse events, including increased mortality [10].

Compared to individuals without behavioural symptoms, those with behavioural symptoms had a longer LOS [56]. However, this review did not find evidence of an association between the total cost of hospital admission and the mean BPSD score [16]. BPSD increased the likelihood of institutionalisation after discharge [35, 56], but we found evidence of poor discharge communication between acute hospitals and care facilities, including the omission of information about BPSD occurrence and care plans [32]. Patients with BPSD were at risk for emergency admissions [42, 46] and hospital readmissions [29, 56]. Additionally, there were positive associations between BPSD and distress among family carers and hospital professionals, although these associations varied depending on the types of BPSD [5, 16, 52] (see Appendix 8 for more details).

## Discussion

To the best of our knowledge, this is the first systematic review and meta-analysis of the prevalence of BPSD of older patients in acute hospital settings. We also synthesised the current evidence on risk factors, treatments, and outcomes for BPSD in acute hospitals. From this review, BPSD was prevalent during acute hospital admissions of older adults with dementia. Poor cognitive functions, pain, and a history of institutionalisation and delirium were major risks for developing BPSD. Over the course of hospitalisation, older patients with BPSD often had insufficient care, including physical restraints, overused psychotropics, and invasive treatments, which could lead to adverse events and death.

### Prevalence of BPSD in acute hospitals

In our review, 60% of older inpatients with dementia in acute hospitals had one or more BPSD symptoms, reflecting the common challenges for hospital care. From our meta-analysis, the major source of heterogeneity of the pooled prevalence of the overall BPSD was the variations of assessment tools for BPSD symptoms of the included studies. From subgroup analysis, we found the higher prevalence of BPSD when assessing by structured assessment tools, e.g. BEHAVE-ad and NPI. The use of assessment tools, rather than clinical interview or medical chart review, could offer a more sensitive and systematic detection and may lead to earlier proper management.

In this review, the pooled prevalence of the individual BPSD ranged from 6% (elation) to 39% (aggression and agitation). Approximately one-third of older inpatients with dementia in the included studies had anxiety, aggression/agitation, apathy, depression, eating problems, irritability, and sleep disorders. Compared to an earlier systematic review of BPSD in community setting [18], the prevalence of individual BPSD in acute hospitals was generally higher, including aggression/agitation (39% [acute setting] vs 27% [community setting]), delusion (24% vs 19%), disinhibition (20% vs 9%), eating problems (36% vs 20%), elation (6% vs 4%), hallucinations (16% vs 12%), irritability (32% vs 27%), sleep disturbances (38% vs 21%), and wandering (24% vs 15%). Older people with these BPSD symptoms tended to be more active and less compliance to home or institutional care. Problems with eating could also cause significant weight loss and physical decline. These could require hospitalisation to ease carer burden and stabilise physical conditions [57, 58]. While we found the prevalence of anxiety (29% [acute setting] vs 29% [community setting]) and depression (28% vs 29%) was relatively similar to the previous systematic review [18], the prevalence of apathy in hospital settings was lower than the prevalence in community settings (27% vs 32%). Apathy, depression and anxiety are more likely to be missed as they are not necessarily perceived to be disruptive or distressing for patients or hospital staff. These symptoms may also pose less challenges for family and professional carers to provide care at home or nursing homes, and people with dementia with these BPSD symptoms may be able to live in their community.

The inconsistent assessment and reporting of delirium across studies may have affected the estimated pooled prevalence of BPSD in acute hospitals. Many studies lacked clarity on formal delirium assessment, limiting sensitivity analysis. Some studies included delirium cases in their BPSD analyses, reflecting the conditions’ co-occurrence and diagnostic challenges in clinical practice, especially retrospective studies. Assessment tools in certain studies referred to pre-admission timeframes, potentially representing BPSD beyond the hospital and delirium context. However, BPSD and delirium often coexist with overlapping manifestations [13], and management may be driven by patient needs rather than definitive diagnosis [59]. In clinical practice, close monitoring can help differentiate these conditions, especially for BPSD strongly related to delirium in dementia patients, such as wandering, sleep problems, affective lability, and perceptual disturbance [12, 13].

### Risk factors of BPSD in acute hospitals

The occurrence of BPSD in acute hospital settings is notably influenced by several critical factors that exacerbate these symptoms and complicate patient care. Our systematic review highlights that BPSD are more prevalent in situations characterised by urgency in people with more severe cognitive impairment. The presence of delirium and unmanaged pain further compounds the severity of these symptoms, suggesting a complex interplay between physical and psychological stressors in hospital environments [43, 60]. Additionally, the quality of interactions between patients and hospital carers plays a pivotal role, where poorer interactions may lead to increased confusion and agitation among patients [6, 61]. These findings underline the necessity for healthcare providers to adopt a multidimensional approach in managing BPSD, emphasising the need for timely and effective pain management, delirium screening, and the improvement of communication strategies between staff and patients.

### Treatment and outcomes for BPSD in acute hospitals

We found that older inpatients with BPSD often experience uncomfortable interventions, such as bladder catheterisation, bed alarms, invasive mechanical ventilation, and physical restraints. Although these measures aim to manage acute symptoms and safeguard patient and staff well-being, they can inadvertently exacerbate or worsen the symptoms of BPSD and cause complications [62–64]. Despite evidence supporting the effectiveness and safety of non-pharmacological treatments for managing BPSD, these strategies are underutilised in acute hospital settings, possibly due to limitations in space and resources, particularly staff availability and time [65]. Both family carers and hospital professionals frequently report distress and pressure when managing older patients with BPSD, highlighting the emotional toll associated with care provision [63, 66]. The high-pressure, structured environment of hospitals often necessitates a reliance on quicker, pharmacological solutions, which are readily available in acute hospitals but associated with increased morbidity and mortality [67].

Additionally, our findings indicate increased LOS among older adults with BPSD, stemming from an intricate interaction between acute physical decline and unmanaged psychological symptoms, complicating treatment and extending hospitalisation. Crucial information regarding BPSD is also frequently lost during transitions to care facilities, reflecting inadequate discharge planning and communication, and resulting in emergency admissions and hospital readmissions [6, 68]. These insights underscore the need for enhanced integration of non-pharmacological interventions, improved support for caregivers, and better continuity of care to improve outcomes for patients with BPSD in acute hospital settings.

### Strengths and limitations

Our systematic review employed a rigorous methodology, including independent double review processes for study screening, selection, and data extraction, complemented by regular team discussions to refine both the review process and data interpretation. The findings of the review were thorough and provided novel, practical insights into the prevalence, risk factors, treatments, and outcomes of BPSD in acute hospital settings. However, there are some limitations to consider when interpreting these results. First, the substantial variability in study populations and measurement tools and the lack of well-defined inclusion/exclusion criteria in certain studies impacted the accuracy of the pooled prevalence estimates. Particularly, the heterogeneity of rating scales across studies makes pooling data challenging. For example, different instruments like BEHAVE-ad, NPI, and the Cohen–Mansfield Agitation Inventory (CMAI) define and measure aggression and agitation inconsistently [69]. Although over 80 tools are globally available to the clinician and researcher to evaluate BPSD, it is extremely hard to establish which instruments are the best [69]; therefore, we need more precise definitions of BPSD for future research and clinical practice.

Additionally, due to the limited information available in the included studies, we were unable to account for all potential variables affecting BPSD prevalence, such as admission conditions, dementia subtypes and stages, presence of delirium, and BPSD treatments. The presence of delirium could also affect the prevalence estimation of BPSD in the previous studies, and this warrants further well-designed studies to explore the influence of delirium and the longitudinal course of the co-existence. Lastly, while we summarised the risk factors, treatments, and outcomes of BPSD in relation to our primary objective of examining prevalence, the evidence on these aspects may not have been explored in depth across the literature.

### Conclusions

Based on our systematic review, behavioural and psychological symptoms (BPSD) are commonly observed in older patients with dementia during acute hospital admissions. These symptoms include aggression/agitation, irritability, anxiety, depression, sleep disturbances, and eating problems. Emergency health conditions, such as pain and delirium, are linked to a higher risk of BPSD. The presence of BPSD not only increases the likelihood of invasive interventions and psychotropic use but also contributes to distress in family caregiver and hospital staff. Furthermore, interactions between patients and hospital staff are often suboptimal, and discharge plans for people with dementia frequently lack comprehensive strategies for managing BPSD. This fragmentation in care can result in repeated emergency admissions and hospital readmissions.

To address these issues, it is crucial for healthcare systems to implement more cohesive and tailored approaches to managing BPSD in older adults with dementia. Improving staff training, enhancing communication with caregivers, and developing integrated discharge plans can significantly reduce the frequency of BPSD and improve overall patient outcomes. Future research should focus on optimising these strategies to ensure better quality of care and more effective management of dementia-related symptoms in hospital settings.

## Supplementary Material

aa-24-1963-File006_afaf013

aa-24-1963-File007_afaf013

aa-24-1963-File008_afaf013

aa-24-1963-File009_afaf013

aa-24-1963-File010_afaf013

aa-24-1963-File011_afaf013

aa-24-1963-File012_afaf013

aa-24-1963-File013_afaf013

## Data Availability

Data will be made available on request.
