# Supplementary material for: Behavioural and psychological symptoms of people with dementia in acute hospital settings: a systematic review and meta-analysis
Source: Age Ageing. 2025 Jan 31;54(1):afaf013. doi: 10.1093/ageing/afaf013 (PMC11784590; doi:10.1093/ageing/afaf013)
Supplement: aa-24-1963-File007_afaf013 [file aa-24-1963-file007_afaf013.pdf]

Study title: Behavioural and psychological symptoms of people with dementia in acute hospital settings: a systematic review and meta-analysis

Appendix 2 Full search strategies

Medline (via Ovid) 05/03/2024

	Ovid MEDLINE(R) and Epub Ahead of Print, In-Process, In-Data-Review & Other Non-Indexed Citations, Daily and Versions <1946 to March 5, 2024>	
1	exp Dementia/	212346
2	dement*.mp.	171839
3	alzheimer*.mp.	211319
4	behavio*ral.mp.	503568
5	psychological.mp.	685940
6	psychiatric.mp.	328973
7	neuropsychiatric.mp.	42749
8	affective.mp.	85789
9	emotion*.mp.	306123
10	mental.mp.	699514
11	depress*.mp.	671295
12	dysphori*.mp.	7734
13	apathy.mp.	6934
14	indifference.mp.	2687
15	anxiet*.mp.	327341
16	anxious.mp.	21170
17	phobi*.mp.	20037
18	elat*.mp.	2963
19	euphori*.mp.	3868
20	irritab*.mp.	32968
21	labil*.mp.	41202
22	agitat*.mp.	27400
23	aggress*.mp.	276930
24	disinhibit*.mp.	10208

Study title: Behavioural and psychological symptoms of people with dementia in acute hospital settings: a systematic review and meta-analysis

25	Impulsi*.mp.	28692
26	hallucinat*.mp.	21865
27	Delusion*.mp.	15924
28	psychos*.mp.	228535
29	Aberrant motor.mp.	403
30	Wander*.mp.	6243
31	sleep.mp.	253792
32	insomnia.mp.	31228
33	appetite.mp.	41155
34	eating.mp.	159331
35	feeding.mp.	353729
36	hospital*.mp.	2025413
37	ward.mp.	48601
38	exp Inpatients/	30684
39	inpatient.mp.	109430
40	1 or 2 or 3	336781
41	4 or 5 or 6 or 7 or 8 or 9 or 10 or 11 or 12 or 13 or 14 or 15 or 16 or 17 or 18 or 19 or 20 or 21 or 22 or 23 or 24 or 25 or 26 or 27 or 28 or 29 or 30 or 31 or 32 or 33 or 34 or 35	3449660
42	36 or 37 or 38 or 39	2083344
43	40 and 41 and 42	8219
44	limit 43 to English language	7171
45	limit 44 to yr="2023 - 2024"	525

Total hits: 7171

Study title: Behavioural and psychological symptoms of people with dementia in acute hospital settings: a systematic review and meta-analysis

PsycInfo (via Ovid) (end of 02/2024)

	APA PsycInfo 1806 to February Week 5 2024	
1	exp Dementia/	94845
2	dement*.mp.	93352
3	alzheimer*.mp.	79426
4	behavio*ral.mp.	440176
5	psychological.mp.	595537
6	psychiatric.mp.	307433
7	neuropsychiatric.mp.	38658
8	affective.mp.	137182
9	emotion*.mp.	504722
10	mental.mp.	692406
11	depress*.mp.	445198
12	dysphori*.mp. [mp=title, abstract, heading word, table of contents, key concepts, original title, tests & measures, mesh word]	8164
13	apathy.mp.	6145
14	indifference.mp.	3236
15	anxiet*.mp.	300790
16	anxious.mp.	25361
17	phobi*.mp.	24383
18	elat*.mp.	1296
19	euphori*.mp. [mp=title, abstract, heading word, table of contents, key concepts, original title, tests & measures, mesh word]	2730
20	irritab*.mp. [mp=title, abstract, heading word, table of contents, key concepts, original title, tests & measures, mesh word]	11620
21	labil*.mp.	4237
22	agitat*.mp. [mp=title, abstract, heading word, table of contents, key concepts, original title, tests & measures, mesh word]	10520
23	aggress*.mp.	103783

Study title: Behavioural and psychological symptoms of people with dementia in acute hospital settings: a systematic review and meta-analysis

24	disinhibit*.mp.	8115
25	Impulsi*.mp.	35436
26	Neurobehavio*.mp. [mp=title, abstract, heading word, table of contents, key concepts, original title, tests & measures, mesh word]	8974
27	hallucinat*.mp.	19259
28	Delusion*.mp.	18216
29	psychos*.mp.	259287
30	Aberrant motor.mp.	285
31	Wander*.mp.	3826
32	sleep.mp.	98012
33	insomnia.mp.	17534
34	appetite.mp.	11592
35	eating.mp.	77594
36	feeding.mp.	52108
37	hospital*.mp.	227238
38	ward.mp.	13336
39	inpatient.mp.	36733
40	exp Hospitalized Patients/	14891
41	1 or 2 or 3	132673
42	4 or 5 or 6 or 7 or 8 or 9 or 10 or 11 or 12 or 13 or 14 or 15 or 16 or 17 or 18 or 19 or 20 or 21 or 22 or 23 or 24 or 25 or 26 or 27 or 28 or 29 or 30 or 31 or 32 or 33 or 34 or 35 or 36	2400208
43	37 or 38 or 39 or 40	248038
44	41 and 42 and 43	6211
45	limit 44 to English language	5645
46	limit 45 to yr="2023 - 2024"	126

Total hits: 5645

Study title: Behavioural and psychological symptoms of people with dementia in acute hospital settings: a systematic review and meta-analysis

Cochrane library (05/03/2024)

	Cochrane library < Cochrane Database of Systematic Reviews Issue 3 of 12, March 2024>	
#1	dement*	31223
#2	alzheimer*	15909
#3	MeSH descriptor: [Dementia] explode all trees	9281
#4	MeSH descriptor: [Alzheimer Disease] explode all trees	5232
#5	behavio*ral	81741
#6	psychological	72278
#7	psychiatric	39028
#8	neuropsychiatric	5464
#9	affective	10886
#10	emotion*	40165
#11	MeSH descriptor: [Mental Disorders] explode all trees	105646
#12	mental	90874
#13	depress*	134990
#14	dysphori*	1324
#15	apathy	1327
#16	indifference	142
#17	anxiet*	78531
#18	phobi*	4393
#19	elat*	403
#20	euphori*	1016
#21	irritab*	9277
#22	labil*	1048
#23	agitat*	7333
#24	aggress*	14056
#25	disinhibit*	860
#26	Impulsi*	3346
#27	hallucinat*	3177

Study title: Behavioural and psychological symptoms of people with dementia in acute hospital settings: a systematic review and meta-analysis

#28	Delusion*	1844
#29	psychos*	37082
#30	Aberrant motor	261
#31	Wander*	688
#32	sleep	54672
#33	appetite	13800
#34	eating	21303
#35	hospital*	436323
#36	ward	19607
#37	inpatient	17298
#38	#1 or #2 or #3 or #4	35576
#39	#5 or #6 or #7 or #8 or #9 or #10 or #11 or #12 or #13 or #14 or #15 or #16 or #17 or #18 or #19 or #20 or #21 or #22 or #23 or #24 or #25 or #26 or #27 or #28 or #29 or #30 or #31 or #32 or #33 or #34	422298
#40	#35 or #36 or #37	448741
#41	#38 AND #39 AND #40<br>in Cochrane Reviews, Cochrane Protocols and Trials	4793
#42	#41 custom range 25/01/23-05/03/24<br>Trials = 262	300

Total hits: 4793

Total citations retrieved: 17609 (Medline 7171, PsycINFO 5645, Cochrane library 4793)

De-duplication: 2508

Citations for title and abstract selection = **15101**
