# Supplementary material for: Behavioural and psychological symptoms of people with dementia in acute hospital settings: a systematic review and meta-analysis
Source: Age Ageing. 2025 Jan 31;54(1):afaf013. doi: 10.1093/ageing/afaf013 (PMC11784590; doi:10.1093/ageing/afaf013)
Supplement: aa-24-1963-File009_afaf013 [file aa-24-1963-file009_afaf013.pdf]

Study title: Behavioural and psychological symptoms of people with dementia in acute hospital settings: a systematic review and meta-analysis

Appendix 4 Study characteristics of the included studies (N=30)

**First Author (year, country)**	**Study aims**	**Study design**	**Study population (dementia diagnostic tools)**	**Sample size**	**Age (Mean (SD)) years**	**Female (%)**	**Admitting conditions and length of stay (LOS)**	**Assessed delirium (delirium prevalence, %)**	**Stage or level of cognitive function**	**No. of BPSD symptoms (assessment tool)**	**Quality rating**
Aminoff, B.Z. (2016, Israel) (49)	Study possible interrelations between not calm and Aminoff suffering syndrome in advanced Alzheimer's disease	Cross-sectional	With advanced dementia (DSM-IV), admitted to a Geriatric-Internal Medicine Ward of a tertiary general hospital	183	Age range: 56-102 years	60.1%	NA	No	FAST: Stage 7c or higher	2 (Mini-Suffering State Examination (MSSE) scale)	Poor
Berish, D. (2024, USA) (29)	1) Examines the prevalence of BPSD during acute hospitalization and 2) examine if BPSD are predictive of adverse patient outcomes	Secondary data analysis of baseline RCT data	Over 65 years with dementia, community dwelling prior to acute hospitalisation	459	81.56 (Range 65–105)	59.0%	NA	CAM-S (60%)	MoCA: Mean (range) = 11.63 (0–25)	4 (NPI)	Good
Boltz, M. (2023, USA) (30)	1) Examine the relationship between delirium severity in patients with dementia upon admission to the hospital and the manifestation of behavioural symptoms; and 2) evaluate the mediating effects of cognitive and physical function, pain, medications, and restraints	Secondary data analysis of baseline RCT data	Over 65 years with dementia, community dwelling prior to acute hospitalization	455	81.5 (8.4)	59.1%	NA	CAM-S (60%)	MoCA: Mean (SD) = 11.7 (7.0), Barthel index: Mean (SD) = 63.6 (27.6)	12 (NPI)	Good

Study title: Behavioural and psychological symptoms of people with dementia in acute hospital settings: a systematic review and meta-analysis

Crowther, G. J. (2017, UK) (38)	Describe the prevalence of formally diagnosed dementia in those over 75 years admitted to the general hospitals	Retrospective chart review	Over 75 years old, admitted electively or as an emergency to the general hospital (any recorded formal diagnosis of dementia)	116	84.3 (5.6)	63.0%	Falls 28%, confusion 15%, infection 7%; LOS= median = 5 days (range 2–87)	Medical notes (11%)	Mild = 30 (26%), moderate = 51 (44%), severe = 9 (8%), unknown = 26 (22%)	4 (documented diagnosis of depression, anxiety, delusions, hallucinations)	Moderate
Drazich, BF. (2023, USA) (31)	1) Describe the prevalence of behavioural and psychological symptoms of dementia in hospitalised older adults living with dementia; and 2) examine the association of physical activity and behavioural and psychological symptoms of dementia among hospitalised older adults living with dementia	Secondary data analysis of baseline RCT data	Over 55 years with dementia, admitted to a medical unit for any medical diagnosis	293	83 (7.95)	63.6%	NA	CAM-S (60%)	Saint Louis University Mental Status Examination: Mean (SD) = 7.68 (6.10)	12 (NPI)	Good
Eriksson, S. (2007, Sweden) (44)	Identify characteristics associated with falls in patients with dementia	Prospective cohort study	With dementia (DSM-IV) in the psychogeriatric ward at an University hospital	204	78.7 (7.4)	61.8%	Conditions= NA; LOS= median = 52.5 days	No	FAST: 87% stage between 6c and 7f	2 (BEHAVE-AD)	Moderate
Ferreira, A. R. (2023, Portugal) (45)	1) Characterize all hospitalizations with dementia-related agitation; and 2) investigate a relationship between agitation and a range of hospitalization outcomes, including length of stay, in-hospital mortality and all-cause hospital readmissions	Retrospective cohort study	Over the age of 65 with dementia (ICD-9-CM), hospitalised from 2010–2015	53156	83.0 (6.72)	62.0%	Pneumonia 19.7%, urinary tract infections 9.0%, septicaemia 6.0%, acute bronchitis 6.0%, acute cerebrovascular disease 5.6%; LOS= NA	Medical notes (4.5%)	NA	1 (ICD-9-CM code of dementia with behavioural disturbance (294.11 and 294.21) and dementia without behavioural disturbance (294.10 and 294.20))	Moderate

Study title: Behavioural and psychological symptoms of people with dementia in acute hospital settings: a systematic review and meta-analysis

Fox, A. (2021, Australia) (55)	Examine the acute hospital experience of older people with and without dementia specifically focusing on length of stay, functional decline, adverse hospital events, and access to allied health therapies	Retrospective chart review	Over the age of 65 years, admitted to an acute care ward for 24 hours or more (ICD)	120	Dementia 83.5 (7.38) vs Without dementia 76.4 (8.27)	Dementia 49.2% vs Without dementia 55.0%	Non-surgical conditions in dementia 85.0% vs non-dementia 70.8%; LOS= patients with dementia (median = 5.0 days) vs patients without (median = 2.2 days)	Medical notes (6.7%)	NA	1 (medical records of level of function including feeding (independent vs not independence))	Moderate
Gilmore-Bykovskyi, A. L. (2021, USA) (32)	1) Assess written discharge communication regarding NPS and associated care management strategies during hospital to SNF transitions; and 2) determine rates of omissions in communication about target NPS and management strategies	Retrospective cohort study	Medicare beneficiaries with dementia (ICD-9), hospitalised due to stroke or hip/femur fracture and discharged to skilled nursing facilities from two urban hospitals	298	84.60 (6.71)	73.5%	Hip fracture 81.2%, stroke 18.8%; LOS= mean (SD) = 6.1 (3.8) days	No	NA	7 (NPI)	Good
Hessler, J. (2018, Germany) (5)	Examine the frequency of BPSD in general hospitals and their associations with nursing staff distress and complications in care.	Cross-sectional study (population based)	65 years or older, admitted on the wards in general hospitals (DSM-IV)	1469	Dementia group 83.25 (7.13) vs Whole sample 78.64 (7.42)	54.1%	NA	CAM (17.8%)	CDR: Mild = 100 (37.0%), moderate = 97 (35.9%), severe = 73 (27.0%)	11 (NPI)	Good
Hwang, J. P. (1996, Taiwan) (50)	1) Describe the frequencies and characteristics of psychotic symptoms in psychiatric inpatients with dementia; and 2) identify the characteristics of dementia with and without psychotic symptoms	Prospective cohort study	With Alzheimer's disease or vascular dementia (DSM-III-R) , admitted to psychiatric ward in a general hospital	78	Alzheimer group 73.9 (7.0) vs Vascular group 75.4 (5.9)	Alzheimer group 42.6% vs Vascular group 4.2%	Most of them were admitted due to violence, inappropriate behaviours and emotional problems; LOS= mean (SD) in Alzheimer group 26.2 (15.4) days vs Vascular group 28.8 (19.7) days	No	MMSE: 9.5 (5.8) for Alzheimer dementia vs 13.4 (6.4) for vascular dementia	1 (BEHAVE-AD)	Moderate

Study title: Behavioural and psychological symptoms of people with dementia in acute hospital settings: a systematic review and meta-analysis

Hwang, J. P. (1997, Taiwan) (51)	Describe the frequency, types and characteristics of behavioural disturbances in psychiatric inpatients with Dementia of the Alzheimer's type	Prospective cohort study	With Alzheimer's disease (DSM-III-R), admitted to the Section of Geriatric Psychiatry of an general hospital	75	74.3 (6.7)	33.3%	NA	No	MMSE (cut-offs = less 10, 11-17, more than 18: Mild = 8 (10.6%), Moderate = 27 (36.0%), Severe = 40 (53.3%)	8 (BEHAVE-AD)	Moderate
Kunik, M. E. (1999, USA) (33)	Examine the association of depression and psychosis with behavioural disturbances in geropsychiatric inpatients with dementia	Retrospective cohort study	All admissions of people with dementia (DSM-III-R and DSM-IV) at the medical centre hospital	208	72 (6.35)	1.96%	NA	No	MMSE, Mean (SD)= 17.64 (7.78)	2 (Hamilton Depression Rating Scale (HDRS), Thought Disorder subscale of the Brief Psychiatric Rating Scale (BPRS))	Moderate
Kupeli, N. (2018, UK) (39)	Explore the factor structure of the CMAI to further validate this tool in a sample of people with dementia during their admission to acute hospital settings	Longitudinal observational cohort study	Over 70 years old with dementia (DSM-IV) and an unplanned acute medical admission	230	Age range: 75-84, n=85; 85-94, n=118; 95+, n=27	65.70%	NA	CAM (excluded delirium cases)	NA	1 (Cohen-Mansfield Agitation Inventory (CMAI))	Moderate

Study title: Behavioural and psychological symptoms of people with dementia in acute hospital settings: a systematic review and meta-analysis

Nandwana, V. (2021, USA) (34)	1) Compare the sociodemographic characteristics and psychiatric comorbidities in Alzheimer's dementia inpatients with manic episodes versus without manic episodes; and 2) evaluate the demographic predictors and risk factors for manic episodes in AD inpatients.	Case-control study	Over 60 years old with Alzheimer's dementia, grouped by a primary discharge diagnosis of a manic episode (diagnostic tool= NA)	34285	75.6 (7.7)	63.80%	NA	No	NA	1 (documented diagnosis of Alzheimer's dementia and manic episode, unspecified)	Moderate
Nourhashemi, F. (2001, France) (46)	Study the underlying reasons for emergency hospital admission of patients with dementia of the Alzheimer type and their characteristics.	Prospective cohort study	With dementia of the Alzheimer type, admitted on an emergency basis (The NINCDS-ADRDA Alzheimer's Criteria)	118	Overall 82.0 (6.9); women 82 (7); men 81.9 (6.6)	72.0%	Behavioural problems 26.3%, falls and/or injury 18.6%, gastrointestinal problems 14.4%, fever 11%, cardiovascular disorders 9.3%, neurological disorders 7.6%, loss of consciousness 4.2%, social grounds 2.5%, others 6.1%; LOS= NA	No	MMSE: Mean (SD)= 10.0 (8.8)	NA (overall) (medical records of reasons for admissions including any behavioural problems)	Poor
Ortoleva Bucher, C. (2016, Switzerland) (47)	1) Find the optimal partition of patient profiles based on HoNOS65+ items as provided by cluster analysis; 2) evaluate the validity, reliability, and clinical interpretation and meaningfulness of the final clustering solution; and 3) describe the characteristics of each profile	Retrospective cohort study	Inpatients with dementia (ICD-10) in acute psychogeriatric wards of a university hospital	542	81.34	BPSD-affective, -functional, -somatic, and -psychotic: 134 (57.5%), 53 (55.8%), 77 (56.2%),	The most frequent causes of admission for patients with dementia (BPSD-affective, -functional, -somatic, and -psychotic): agitation (30.9%, 32.6%, 24,8%, and 36.4%), effective (or risk of) harm (28.8%, 24.4%, 32.8%, and 37.7%), and sheltering (16.3%, 24.2%, 16.1%, and 16.9%); LOS= median 34, 41, 43, and 45 days	No	NA	4 (Health of the Nation Outcome Scale for elderly people (HoNOS65+))	Good

Study title: Behavioural and psychological symptoms of people with dementia in acute hospital settings: a systematic review and meta-analysis

						51 (66.2%)					
Pitkala, K. H. (2004, Finland) (48)	1) Describe the prevalence of various psychiatric and behavioural symptoms in patients with dementia in nursing homes and acute geriatric wards; and 2) investigate the administration of psychotropic medications to these patients and their relationship to psychiatric symptoms	Cross-sectional study	Over 70 years old in six acute geriatric wards in two city hospitals and seven nursing homes (DSM-IV)	230	86.1	85.5%	Acute infections 20.4%, falls and fractures 19.4%, cardiovascular diseases 19.0%, psychiatric or neurological problems 6.9%, postoperative rehabilitation 4.6%, or general frailty 12.9%, and patients in terminal care 2.3%; LOS= NA	DSM-IV (43.2%)	NA	12 (self-developed questionnaire with extensive patient interview, various mental tests and collection of data from medical records and caregivers' and nurses' interviews)	Good
Rabins, P. V. (1991, USA) (35)	Review the two-year experience of a single acute psychiatric hospital in caring for persons with dementia.	Retrospective chart review	Individuals discharged with a diagnosis of irreversible dementia (DSM-III-R)	121	NA	56.2%	Agitation 55%, depressive symptoms 42%, hallucinations and/or delusions 33%, sleep disorder 26%, assessment of cognitive change 26%, weight loss or food refusal 22%; LOS= mean 18.6 (12.3) days, median 14.0 days	Medical notes (33% on admission and 11.4% during admission)	NA	6 (medical records of reasons for admission including BPSD)	Moderate

Study title: Behavioural and psychological symptoms of people with dementia in acute hospital settings: a systematic review and meta-analysis

Sampson, E. L. (2014, UK) (16)	1) Define the prevalence and subtypes of BPSD and explore their clinical associations; and 2) explore associations between BPSD (including subtypes) and quality of care, length of stay, adverse events, discharge destination, mortality and costs of the hospital admission	Longitudinal cohort study	Over 70 years old with an unplanned acute medical admission and diagnosis of dementia (DSM-IV)	230	Mean (SD)= 87.2 (5.9); Age range: 75-84, n= 85; 85-94, n= 118; 95+, n= 27	66.0%	Pneumonia/ chest infection 26.7%, urinary tract infection 15.7%, fall or fracture 11.3%, cardiac events 9.6% At the initial study assessment (within 72 h of admission), 11.4% had delirium; LOS= median 12 days (range= 2–72; IQR= 7–23)	CAM (excluded delirium cases)	FAST: Stage 3–5=37.4%, 6a–6c= 17.0%, 6d–6e= 32.2%, 7a–f= 13.5%	7 (BEHAVE-AD)	Good
Sampson, E. L. (2015, UK) (40)	1) Investigate the prevalence of pain in people with dementia admitted to general hospitals; and 2) explore the association between pain and BPSD	Longitudinal cohort study	Over 70 years old with dementia (DSM-IV) and unplanned medical admissions	230	Age range: 75-84, n= 85; 85-94, n = 118; 95+, n= 27	66.0%	Infections (lung/skin/viral) 34%, infections (UTI/blocked catheter) 16%, fall/ fracture/ pain 14%, cardiac 10%, others 26%; LOS= median 12 days (range= 2–72; IQR= 7–23)	CAM (excluded delirium cases)	FAST: Stage 3–5=37.4%, 6a–6c= 17.0%, 6d–6e= 32.2%, 7a–f= 13.5%	7 (BEHAVE-AD, CMAI)	Good
Shah, A. (1995, UK) (41)	Investigate violence in the subsample of patients with dementia	Retrospective study	Patients with dementia (ICD-9), admitted to 60-bedded psychiatric unit attached to a teaching hospital	67	Violent 79.5 (60-92) vs Non-violence 81 (65-97)	64.2%	NA	No	NA	1 (standard incident forms for violence, completed by the nursing staff)	Poor

Study title: Behavioural and psychological symptoms of people with dementia in acute hospital settings: a systematic review and meta-analysis

Sommerlad, A (2019, UK) (42)	1) Describe general hospital admission rates within the national health care provider in people with dementia diagnosed in secondary mental healthcare services; 2) compare admission rates with an age-standardised control population without dementia; and 3) identify factors associated with hospitalisation	Retrospective cohort study	Over 65 years old with dementia (ICD-10) and general, nonpsychiatric admissions; emergency (unplanned) and elective (planned)	7693	People with dementia admitted to hospitals 82.1 (7.0) vs All people with dementia 82.1 (7.2)	60.6%	Of 35,716, emergency admissions 71.8% vs elective admissions 28.2%; LOS= NA	No	MMSE: Mean (SD)= 18.6 (SD 6.2)	4 (Health of the Nation Outcome Scales (HoNoS))	Good
Spears, C. (2019, USA) (36)	Identify common causes, complications, medication profiles, and outcomes of hospitalization for individuals with Lewy body dementia	Retrospective cohort study	People with Lewy body dementia (ICD-9 and ICD-10), admitted to an academic medical centre	178	78 (8, 58-97)	41.0%	Hallucinations or confusion 40%, falls 24%, infection 23%, gastrointestinal disease 7%, cardiac disease 6%, respiratory illness 5%, failure to thrive, failure to cope 5%, genitourinary disorder 3%, elective surgery 3%, intracranial haemorrhage 2%, planned admission (non-surgical) 2%, syncope 1%, stroke 1%, others 13%; LOS= NA	Medical notes (49%)	NA	1 (medical records of reasons for admission including hallucinations or confusion)	Poor

Study title: Behavioural and psychological symptoms of people with dementia in acute hospital settings: a systematic review and meta-analysis

Tan, L. L. (2005, Singapore) (52)	1) Determine the neuropsychiatric symptoms, demographic characteristics and referral patterns of patients with dementia managed in the outpatient setting compared with those admitted to the acute psychogeriatric wards; and 2) assess the impact of neuropsychiatric symptoms on distress in the family and professional caregivers	Cross-sectional study	Patients with dementia (DSM-IV) at the outpatient clinics and acute psychogeriatric wards of a general hospital	42	76.14 (7.36)	52.0%	NA	No	CMMSE: Mean (SD) = 10.07 ± 7.98	12 (NPI)	Good
Tannenbaum, R. (2022, USA) (56)	1) Identify the prevalence of behavioural symptoms in hospitalised people with dementia; 2) determine patient characteristics and hospital practices associated with behavioural symptoms; and 3) explore the association between behavioural symptoms and clinical outcomes	Cross-sectional study	Over 65 years old, admitted to one of seven health system hospitals (ICD-9)	8637)	84.5 (7.7)	61.6%	NA; LOS= median 5.0 days (IQR= 3–8)	Medical notes (43.1%)	NA	NA (overall) (medical records of documented behavioural symptoms and surrogate markers including as-needed dose of antipsychotics or benzodiazepines with an indication for behavioural symptoms, physical restraints or constant)	Moderate
Timmons, S. (2015, Ireland) (37)	1) Investigate differences in dementia prevalence between hospital types, acute and elective admissions, and admitting specialities; and 2) identify predictors and associations of dementia	Cross-sectional study	All people aged ≥70 years admitted for elective and emergency admissions (SMMSE, IQCODE, CAM, and expert diagnosis)	598	Dementia group 84 (7.0) vs Non-dementia 78.3 (5.8)	51.0%	Acute 81.2% vs elective 18.8% Pneumonia accounted for 23.6% of all acute medical admissions in people with dementia; LOS= NA	CAM and DRS-R98 (57%)	CDR: Mild = 55%, moderate = 29%, severe = 16%	1 (Screening question, Geriatric Depression Scale (GDS) or Cornell Scale for Depression in Dementia (CSDD) if MMSE <15/30)	Good

Study title: Behavioural and psychological symptoms of people with dementia in acute hospital settings: a systematic review and meta-analysis

Tsai, S. J. (1997, Taiwan) (53)	Investigate the frequency and the characteristics of delusional jealousy in patients with dementia	Cross-sectional study	People aged 65 and over with dementia (DSM-III) admitted to the psychogeriatric ward	133	Jealousy group 74.1 (5.9) vs Non-jealousy 74.1 (6.3)	26.3%	NA	No	MMSE: Mean (SD) in jealousy group = 13.5 (5.6) vs non-jealousy = 11.3 (6.3)	2 (BEHAVE-AD)	Poor
White, N. (2017, UK) (10)	Describe the pharmacological and non-pharmacological management of BPSD in people with dementia over the age of 70 years who had undergone unplanned acute hospital admission	Longitudinal cohort study	Over 70 years old with dementia and unplanned medical admissions (CAM to exclude delirium, then MMSE and DSM-IV)	230	Mean (SD)= 87.2 (5.9); Age range: 75-84, n= 85; 85-94, n= 118; 95+, n= 27	65.7%	Infection - lungs/skin/viral 34.5%, infection - UTI/blocked catheter 15.7%, fall/fracture/pain 31 13.5%, cardiac 9.6%, others 26.7%; LOS= NA	CAM (excluded delirium cases)	FAST: Stage 3–5=37.4%, 6a–6c= 17.0%, 6d–6e= 32.2%, 7a–f= 13.5%	7 (BEHAVE-AD)	Good
Yang, H. (2020, China) (54)	1) Provide new prevention and treatment ideas for clinical prevention and treatment of depressive symptoms in patients with Alzheimer's disease; and 2) analyse risk factors associated with depressive symptoms in patients with Alzheimer's diseas	Cross-sectional study	Inpatients with Alzheimer's disease at the Department of Neurology at the medical university hospital (The NINCDS-ADRDA Alzheimer's Criteria)	158	With depression 74.4 (5.7) vs Without depression 73.6 (5.9)	53.2%	NA	No	MMSE: Mean (SD) in Alzheimer group with depression = 13.9 (7.7) vs without depression = 15.2 (7.5)	1 (Hamilton Depression Rating Scale (HDRS))	Poor
