# Supplementary material for: Behavioural and psychological symptoms of people with dementia in acute hospital settings: a systematic review and meta-analysis
Source: Age Ageing. 2025 Jan 31;54(1):afaf013. doi: 10.1093/ageing/afaf013 (PMC11784590; doi:10.1093/ageing/afaf013)
Supplement: aa-24-1963-File010_afaf013 [file aa-24-1963-file010_afaf013.pdf]

Study title: Behavioural and psychological symptoms of people with dementia in acute hospital settings: a systematic review and meta-analysis

Appendix 5 Quality assessment of the included studies (N=30) using Joanna Briggs Institute (JBI) Critical Appraisal Checklist for Studies Reporting Prevalence Data to assess the quality of eligible studies (Munn et al., 2015)

Authors (year)	1st item	2nd item	3rd item	4th item	5th item	6th item	7th item	8th item	9th item	Overall judgment
Aminoff, B. Z. (2016)	Yes	Yes	Unclear	Yes	Yes	Yes	Unclear	No	No	Poor
Berish, D. (2024)	Yes	Yes	Yes	Yes	Yes	Yes	Yes	Yes	Unclear	Good
Boltz, M. (2023)	Yes	Yes	Yes	Yes	Yes	Yes	Yes	Yes	Unclear	Good
Crowther, G. J. (2017)	Yes	Yes	Yes	Yes	Yes	No	No	Yes	NA	Moderate
Drazich, BF. (2023)	Yes	Yes	Yes	Yes	Yes	Yes	Yes	Yes	Unclear	Good
Eriksson, S. (2007)	Yes	Yes	Unclear	Yes	Yes	Yes	Yes	Yes	NA	Moderate
Ferreira, A. R. (2023)	Yes	Yes	Unclear	Yes	Yes	Yes	Yes	No	Yes	Moderate
Fox, A. (2021)	Yes	Yes	Yes	Yes	Yes	No	No	Yes	NA	Moderate
Gilmore-Bykovskyi, A. L. (2021)	Yes	Yes	Yes	Yes	Yes	Yes	Yes	Yes	NA	Good
Hessler, J. (2018)	Yes	Yes	Unclear	Yes	Yes	Yes	Yes	Yes	Yes	Good
Hwang, J. P. (1996)	Yes	Yes	Unclear	Yes	Yes	Yes	Yes	No	No	Moderate
Hwang, J. P. (1997)	Yes	Yes	Unclear	Yes	Yes	Yes	Yes	Unclear	No	Moderate
Kunik, M. E. (1999)	Yes	Yes	Unclear	Yes	Yes	Yes	Yes	No	No	Moderate
Kupeli, N. (2018)	Yes	Yes	Yes	No	Yes	Yes	Yes	Yes	No	Moderate
Nandwana, V. (2021)	Yes	Yes	Yes	Yes	Yes	No	No	No	Unclear	Moderate
Nourhashemi, F. (2001)	Yes	Unclear	Unclear	Yes	Unclear	No	No	Yes	Yes	Poor
Ortoleva Bucher, C. (2016)	Yes	Yes	Yes	Yes	Yes	Yes	Unclear	Yes	Yes	Good
Pitkala, K. H. (2004)	Yes	Yes	Yes	Yes	Yes	Yes	Unclear	Yes	Yes	Good
Rabins, P. V. (1991)	Yes	Yes	Unclear	No	Yes	Unclear	Yes	Yes	Yes	Moderate
Sampson, E. L. (2014)	Yes	Yes	Yes	Yes	Yes	Yes	Yes	Yes	Yes	Good
Sampson, E. L. (2015)	Yes	Yes	Yes	Yes	Yes	Yes	Yes	Yes	Yes	Good
Shah, A. (1995)	Yes	Yes	No	Yes	Unclear	No	No	Yes	Unclear	Poor
Sommerlad, A (2019)	Yes	Yes	Yes	Yes	Yes	Yes	Yes	Yes	NA	Good
Spears, C. (2019)	Yes	Yes	Yes	Yes	Unclear	No	No	Yes	Unclear	Poor
Tan, L. L. (2005)	Yes	Yes	Unclear	Yes	Yes	Yes	Yes	Yes	Yes	Good
Tannenbaum, R. (2022)	Yes	Yes	Yes	No	Yes	No	No	Yes	Yes	Moderate
Timmons, S. (2015)	Yes	Yes	Unclear	Yes	Yes	Yes	Yes	Yes	Yes	Good
Tsai, S. J. (1997)	Yes	Yes	Unclear	Yes	Yes	Yes	Unclear	No	Yes	Poor
White, N. (2017)	Yes	Yes	Yes	Yes	Yes	Yes	Yes	Yes	Yes	Good
Yang, H. (2020)	Yes	Yes	Unclear	Yes	Yes	No	Yes	Yes	Unclear	Poor

Reference

Munn, Z., Moola, S., Lisy, K., Riitano, D., & Tufanaru, C. (2015). Methodological guidance for systematic reviews of observational epidemiological studies reporting prevalence and cumulative incidence data. *Int J Evid Based Healthc*, *13*(3), 147-153. https://doi.org/10.1097/XEB.0000000000000054
