# Supplementary material for: Behavioural and psychological symptoms of people with dementia in acute hospital settings: a systematic review and meta-analysis
Source: Age Ageing. 2025 Jan 31;54(1):afaf013. doi: 10.1093/ageing/afaf013 (PMC11784590; doi:10.1093/ageing/afaf013)

Study title: Behavioural and psychological symptoms of people with dementia in acute hospital settings: a systematic review and meta-analysis

Appendix 6 Sensitivity analysis for delirium

Sensitivity analysis	Overall proportion	95% Confidence Interval	I^2 (%)	τ^2
All studies (random-effects model)	0.60	0.43-0.78	100%	0.0874
Excluding a study that not include people with delirium*	0.59	0.40-0.78	100%	0.0947

- *Excluded Sampson EL et al. (2014), also for Kupeli N et al. (2018), Sampson EL et al. (2015) and White N et al. (2017) (four papers, a same study)
- This change indicates that the results are stable, as even with the exclusion of data, the overall proportion changes only slightly. The confidence interval still largely covers the original range, and τ^2 (tau-squared), which measures the variance between studies, shows a small increase.
- This suggests that the results of this study are not significantly affected by the exclusion of data.

Study title: Behavioural and psychological symptoms of people with dementia in acute hospital settings: a systematic review and meta-analysis

- **All studies (random-effects model)**

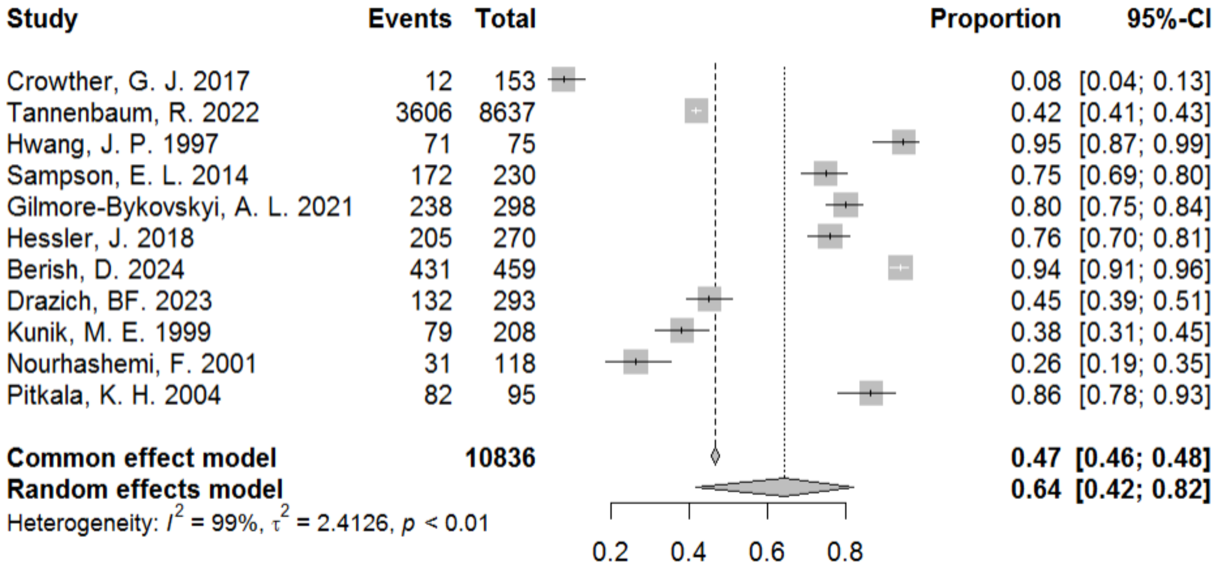

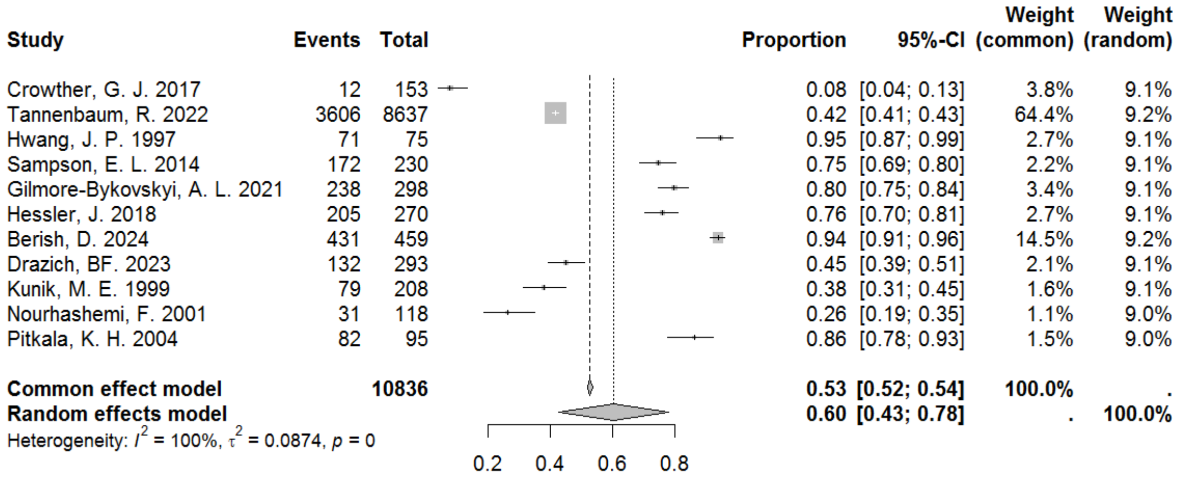

Study title: Behavioural and psychological symptoms of people with dementia in acute hospital settings: a systematic review and meta-analysis

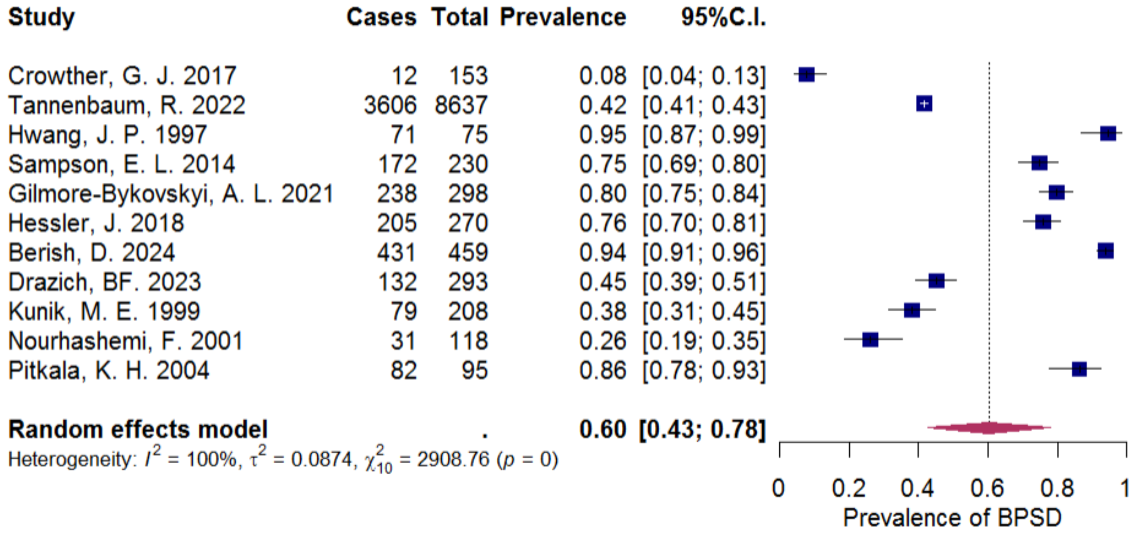

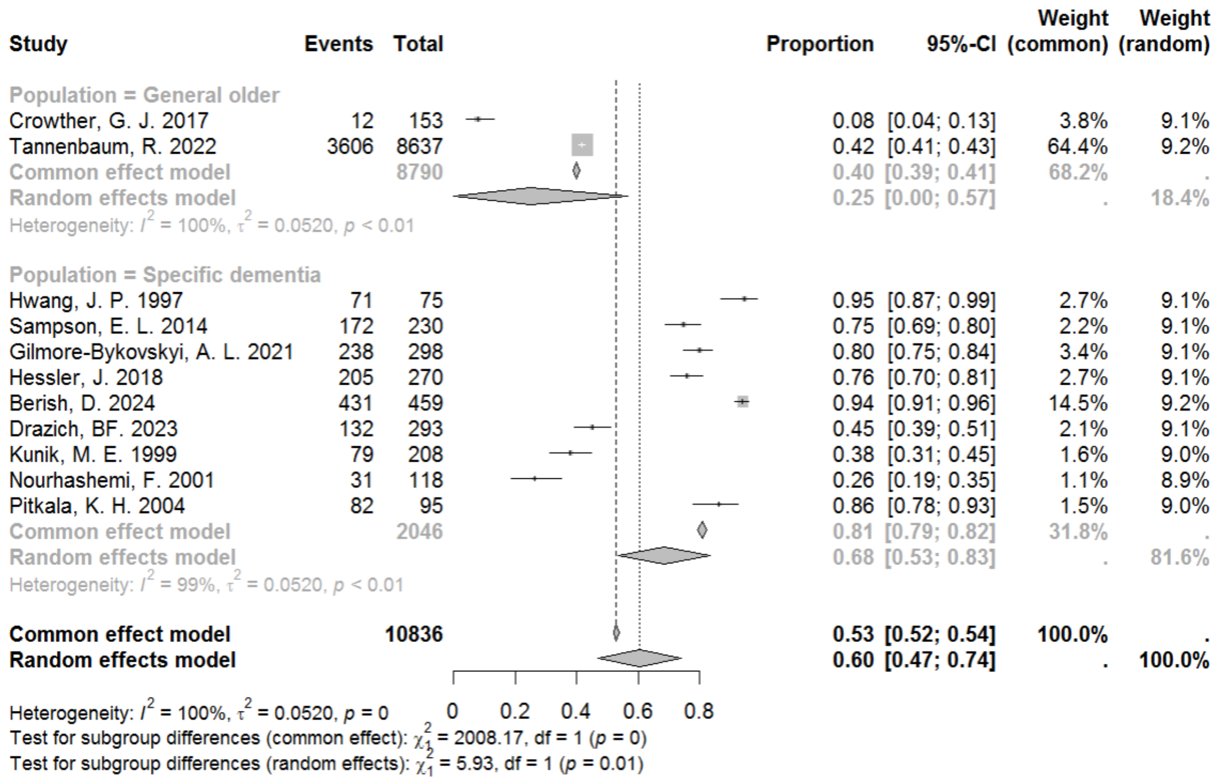

Study title: Behavioural and psychological symptoms of people with dementia in acute hospital settings: a systematic review and meta-analysis

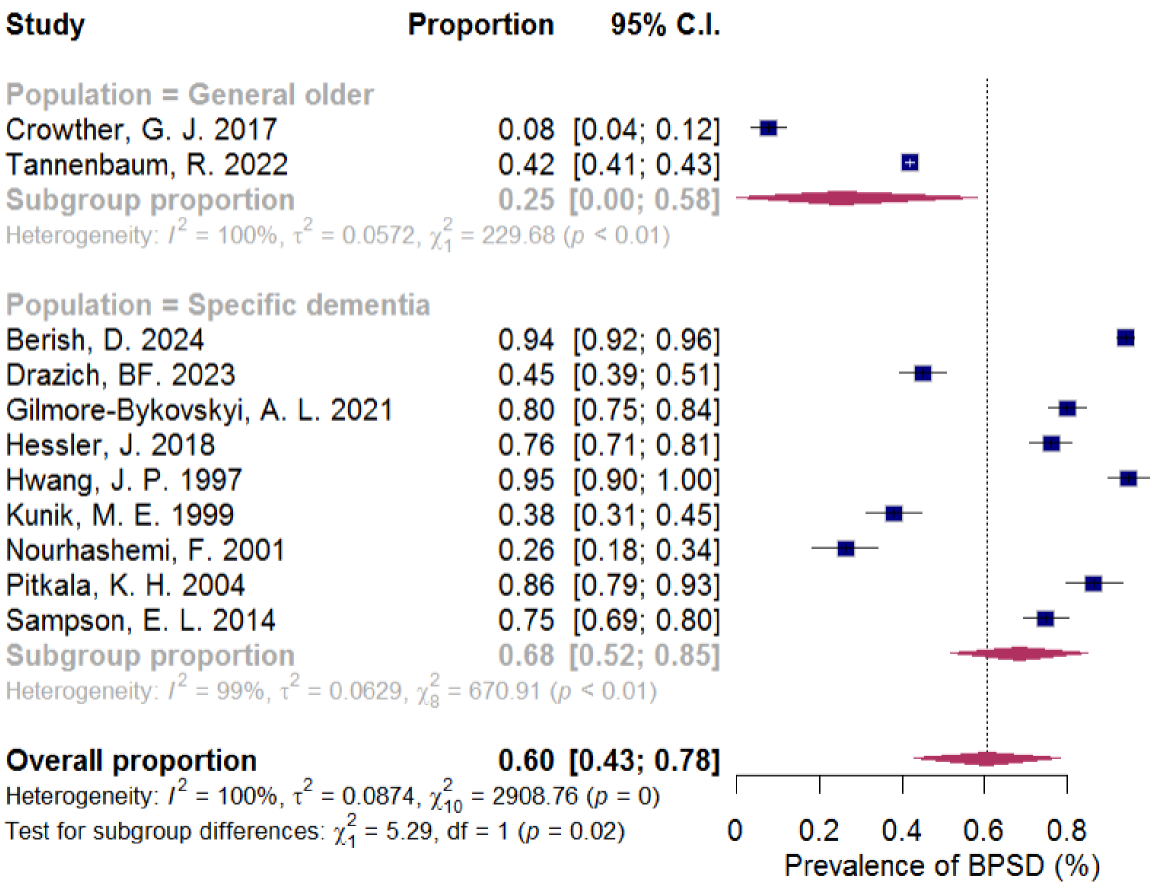

Study title: Behavioural and psychological symptoms of people with dementia in acute hospital settings: a systematic review and meta-analysis

- **Excluded a paper with delirium exclusion**

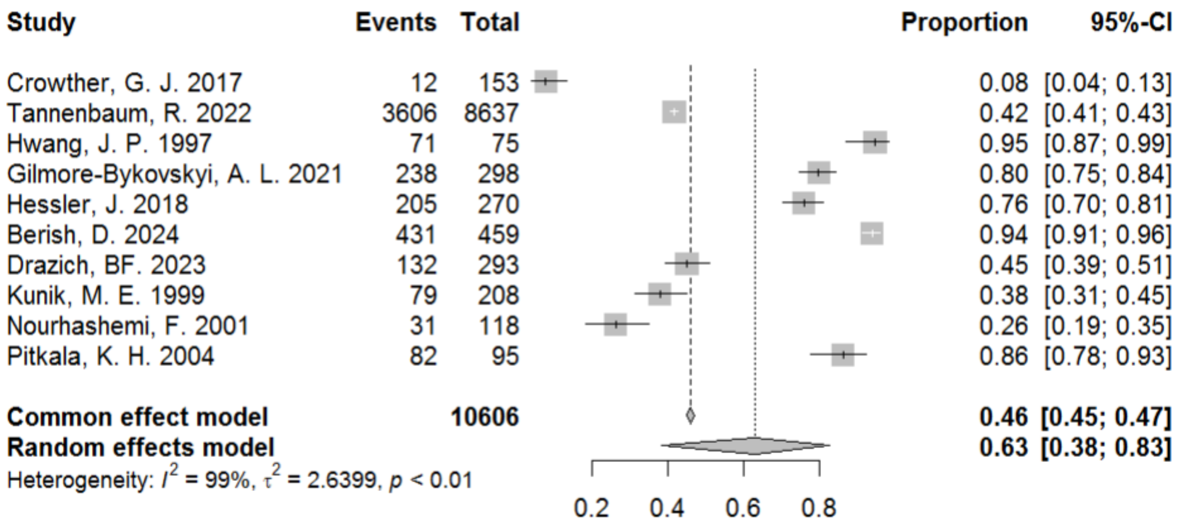

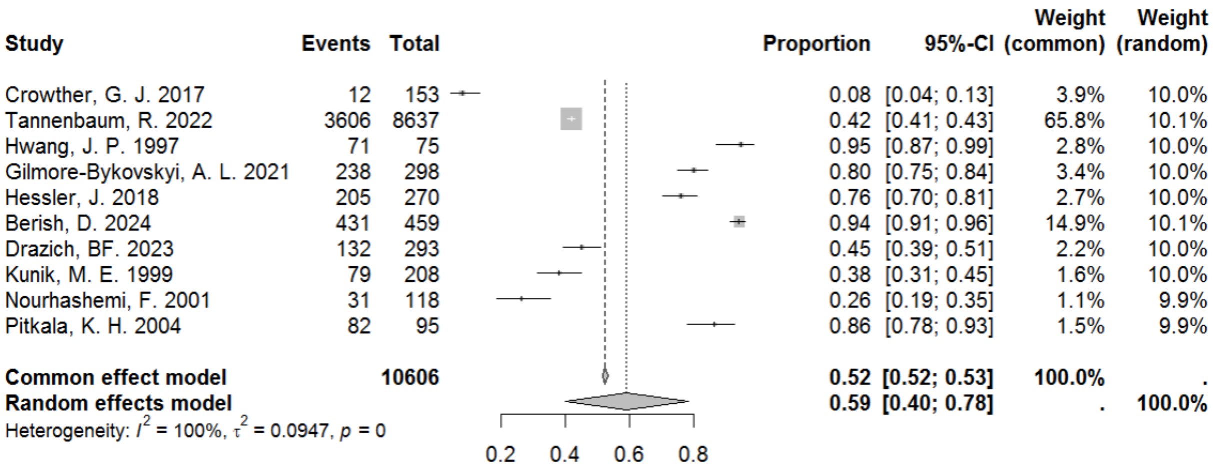

Study title: Behavioural and psychological symptoms of people with dementia in acute hospital settings: a systematic review and meta-analysis

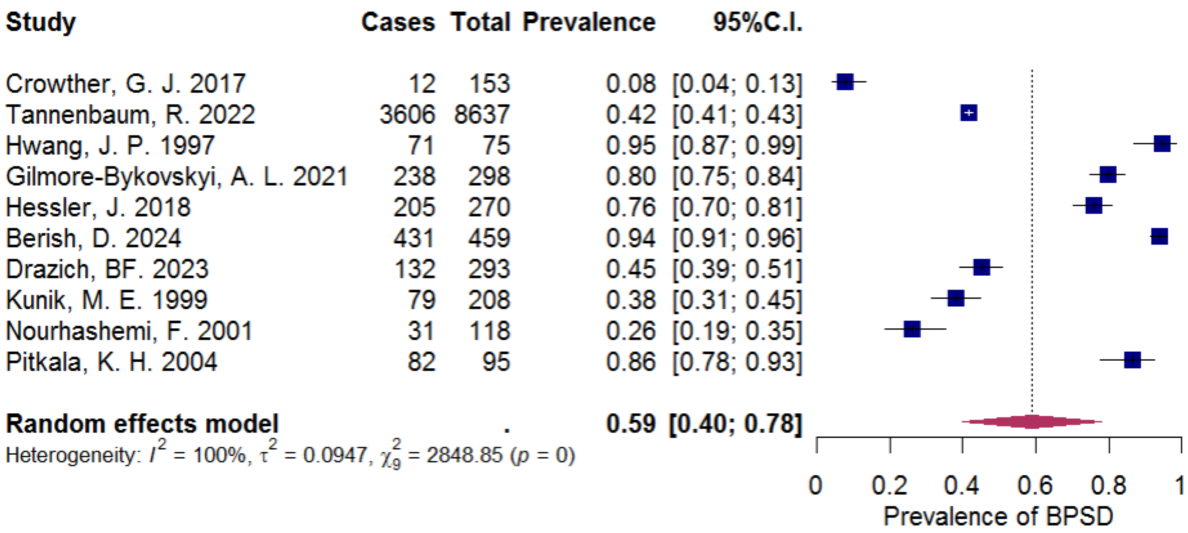

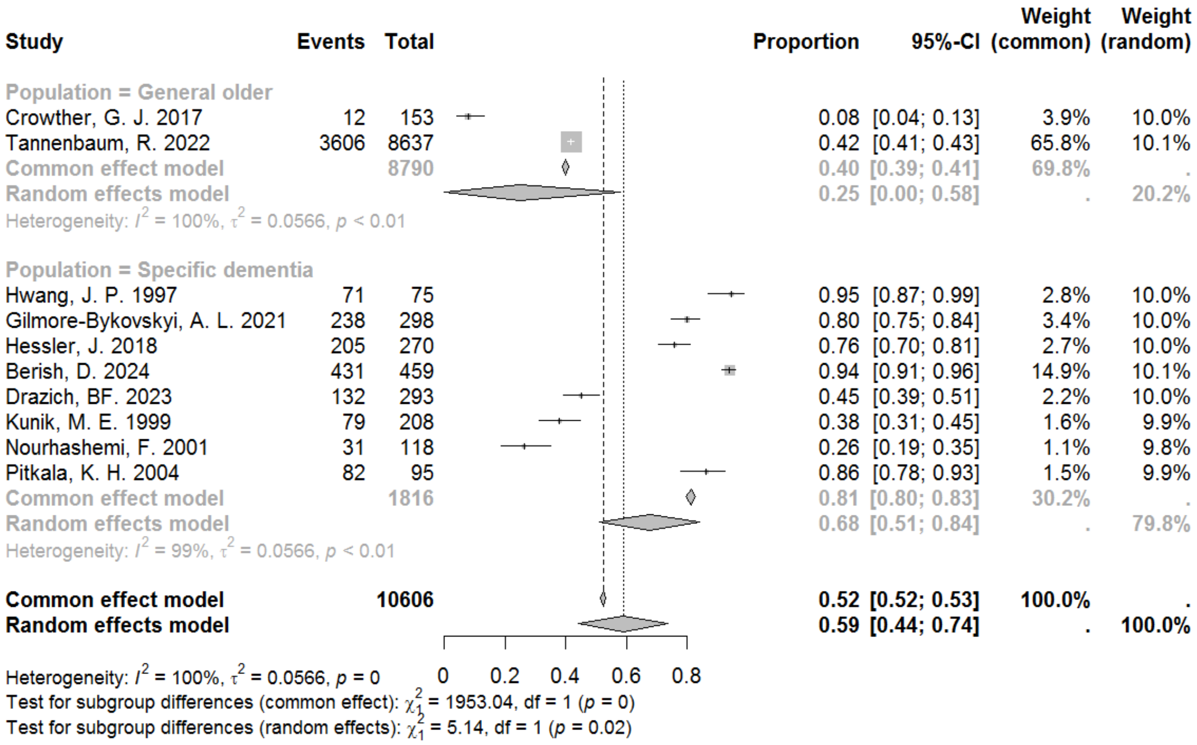

Study title: Behavioural and psychological symptoms of people with dementia in acute hospital settings: a systematic review and meta-analysis

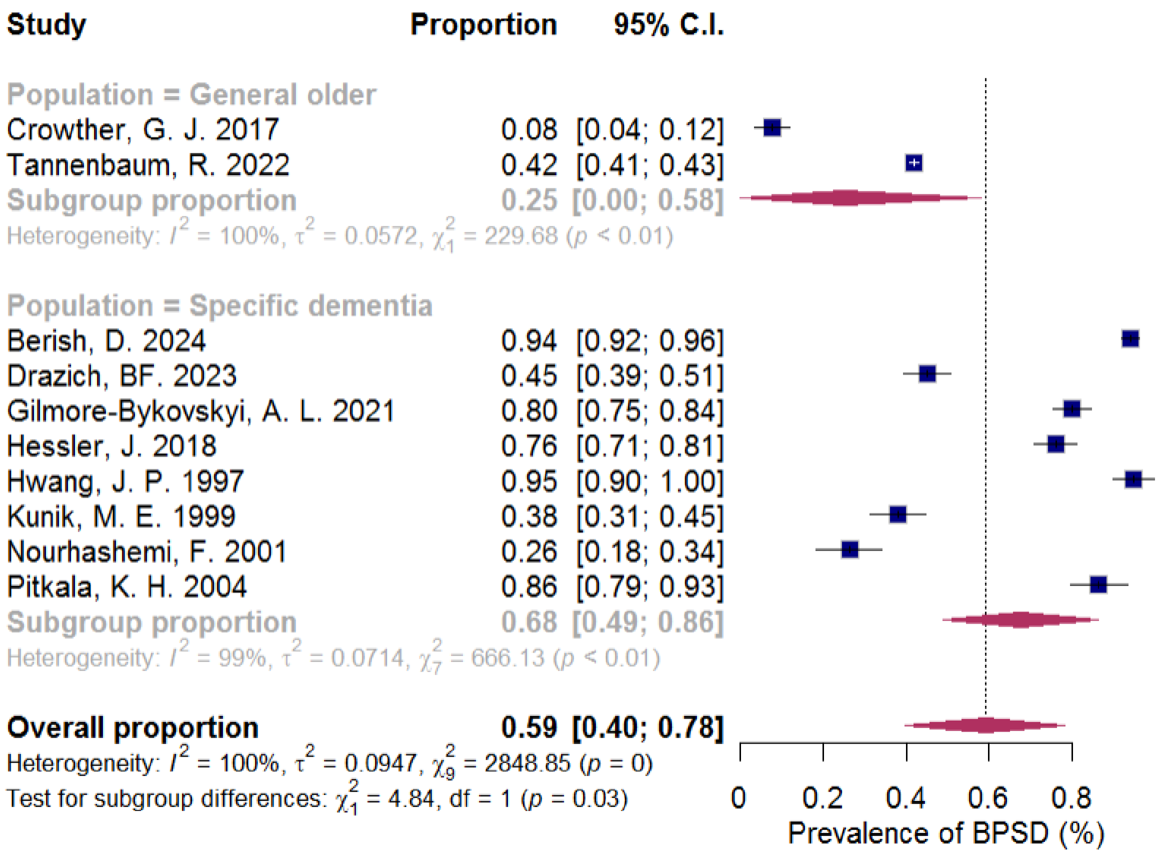

Supplement: aa-24-1963-File011_afaf013 [file aa-24-1963-file011_afaf013.pdf]
