# Supplementary material for: Behavioural and psychological symptoms of people with dementia in acute hospital settings: a systematic review and meta-analysis
Source: Age Ageing. 2025 Jan 31;54(1):afaf013. doi: 10.1093/ageing/afaf013 (PMC11784590; doi:10.1093/ageing/afaf013)
Supplement: aa-24-1963-File012_afaf013 [file aa-24-1963-file012_afaf013.pdf]

Study title: Behavioural and psychological symptoms of people with dementia in acute hospital settings: a systematic review and meta-analysis

Appendix 7 Summary of risk factors of BPSD in acute hospitals reported in the included studies

**Domains of risk factors reported in the included studies**	**Demographics**	**Living arrangements**	**Stage and cognitive levels**	**Admitting conditions**	**Physical health**	**Co-presence of delirium**	**Pain**	**Process of care**	**Interactions between BPSDs**	**Carer characteristics**
Aminoff, B. Z. (2016)					**O**					
Berish, D. (2024)										**O**
Boltz, M. (2023)						**O**				
Drazich, BF. (2023)					**O**		**O**	**O**		
Ferreira, A. R. (2022)	**O**			**O**						
Hessler, J. (2018)			**O**							
Hwang, J. P. (1996)	**O**									
Hwang, J. P. (1997)			**O**							
Kunik, M. E. (1999)			**O**						**O**	
Kupeli, N. (2018)							**O**			
Nandwana, V. (2021)	**O**				**O**				**O**	
Rabins, P. V. (1991)			**O**							
Sampson, E. L. (2014)	**O**	**O**				**O**				
Sampson, E. L. (2015)							**O**			
Tannenbaum, R. (2022)	**O**	**O**		**O**						
Timmons, S. (2015)						**O**				
Tsai, S. J. (1997)			**O**							
Yang, H. (2020)					**O**					

Study title: Behavioural and psychological symptoms of people with dementia in acute hospital settings: a systematic review and meta-analysis

More detailed summary of risk factors associated with BPSD in acute hospital setting

**Domains**	**Risk factors of BPSD (First author, year)**
Demographics	Patients with behavioural symptoms likely to be male (40.3% vs. 36.9%, p = .001) and White (62.7% vs. 58.3%, p < .001). (Tannenbaum, R. (2022))<br>BPSD were more common in men (82.3% vs 70.9%, P=0.078). (Sampson, E. L. (2014))<br>Patients with agitation were significantly younger than their counterparts without agitation (mean 81.19 vs.83.29 years, p<0.001). (Ferreira, A. R. (2022))<br>Patients with psychotic symptoms were older than those without (p=0.037). (Hwang, J. P. (1996))<br>A higher proportion of inpatients with dementia and manic episodes were females (63.8%), Whites (85.2%). Higher-income percentile was at a lower risk for manic episodes (OR: 0.51; 95% CI 0.46-0.57). (Nandwana, V. (2021))
Living arrangements	BPSD were more common in those admitted from residential or nursing homes. (Sampson, E. L. (2014))<br>Patients with behavioural symptoms were more likely to come from a facility (26.6% vs. 23.7%, p < .05). (Tannenbaum, R. (2022))
Stage and cognitive levels	Number of behavioural problems was significantly associated with severity of cognitive impairment (correlation coefficient= -0.279). Wandering (correlation coefficients= -0.362), hyperphagia (correlation coefficients= -0.294) and sleep disturbance (correlation coefficients= -0.272) were associated with severity of cognitive impairment (p<0.05). (Hwang, J. P. (1997))<br>Dementia severity accounted for significant variance in CMAI scores and was positively associated with behavioural disturbance. (Kunik, M. E. (1999))<br>Aberrant motor behaviour, anxiety, aggression and at least one BPSD were statistically significantly associated with dementia severity. (Hessler, J. (2018))<br>At this level greater cognitive impairment is associated with the presence of agitation and the need for more nursing care (i.e. need for assistance with toileting or being fully dependent in dressing, bathing, and feeding). (Rabins, P. V. (1991))<br>Delusional jealousy can develop during any stage of dementia, suggesting different psychobiological factors between delusional jealousy and cognitive function. (Tsai, S. J. (1997))
Admitting conditions	Behavioural symptoms were more likely in those with admission to medicine service (vs. surgical service, 92.7% vs. 91.0%, p= .003). (Tannenbaum, R. (2022))<br>Agitation occurred in the context of an urgent admission (93.2% vs. 6.8% for planned admissions). (Ferreira, A. R. (2022))

Study title: Behavioural and psychological symptoms of people with dementia in acute hospital settings: a systematic review and meta-analysis

Physical health	Physical activity was not associated with BPSD (b = 0.01; p = 0.56), controlling for gender, comorbidities, cognitive impairment severity, age, functional ability, pain, delirium, and the quality of staff-patient interactions. (Drazich, BF. (2023))<br>Compared to those without depression, people with Alzheimer's disease and depression had higher odds of T2DM (OR: 2.18, 95% CI: 1.61–2.54, p=0.005), hypertension (OR: 1.70, 95% CI: 1.45–2.03, p=0.038), and WML (OR: 1.77, 95% CI: 1.39–2.16. p=0.021) (T2DM had the highest risk factor). People with Alzheimer's disease with HbA1c value of 6.5% < HbA1c <\= 7% and HbA1c > 7% had significantly higher depression scale scores than people with Alzheimer's disease with HbA1c <\= 6.5%. (Yang, H. (2020))<br>Patients who were 'Not calm' had more pain (45.2% vs 23.8%, p=0.002), had an unstable medical condition (56.1% vs 22.8%, p=0.0001), fever (51.2% vs 30.7%, P=.005), high WBC (11,176 + 5341 vs 9,237 + 3301, p=0.003) and CRP levels (82.2 + 78.1 vs 56.5 + 56.7, p=0.020); but fewer had history of CVA (25.6% vs 42.6%, p=0.017). (Aminoff, B. Z. (2016))<br>Higher proportion of comorbid tobacco use (5.3% vs. 3.4%, p= 0.031) and cannabis use (1.4% vs. 0%, p= <0.001). (Nandwana, V. (2021))
Co-presence of delirium	BPSD were more common in people presenting with delirium on admission (92.3% vs 72.6%, p=0.030). (Sampson, E. L. (2014))<br>A higher severity of delirium superimposed upon dementia was associated with lower physical and cognitive function, which, in turn, was associated with greater manifestations of BPSD. (Boltz, M. (2023))<br>Delirium was considerably more common in those with dementia, 57% with dementia had delirium on admission, compared with 7% of controls (P < 0.05) (Timmons, S. (2015))
Pain	There was significant association between the total BEHAVE-AD score and pain at movement (p=0.002) and at rest (p=0.003) (PAINAD). (Sampson, E. L. (2015))<br>Pain (b=0.16, p=0.02) was associated with BPSD. (Drazich, BF. (2023))<br>Correlations between the subscales of the CMAI and PAINAD demonstrated significant associations between physically aggressive behaviours and pain during activity (correlation = .15, p<0.05) and verbally aggressive behaviours correlated both with pain at rest (.22, p<0.001) and during activity (.19, p<0.001). (Kupeli, N. (2018))
Process of care	Quality of staff-patient interactions (b= -0.17, p=0.007) was associated with BPSD. (Drazich, BF. (2023))
Interactions between BPSDs	Psychotic symptoms were associated with aggressive behavioural symptoms (p<0.001). (Kunik, M. E. (1999))<br>Inpatients with dementia and manic episodes had higher risk of presenting with suicidal behaviours (OR 1.88; 95% CI 1.23-2.86). (Nandwana, V. (2021))
Carer characteristics	Care partner education level had significant positive associations with several adverse patient outcomes including ER admissions (rho = 0.164, p = .003), hospitalizations (rho = 0.124, p = .023), injuries at 6-month follow-up (rho = 0.123, p

	= .025), falls (rho = 0.149, p = .005), injuries at 2-month follow-up (rho = 0.156, p = .002). (Berish, D. (2024))
