# Supplementary material for: Behavioural and psychological symptoms of people with dementia in acute hospital settings: a systematic review and meta-analysis
Source: Age Ageing. 2025 Jan 31;54(1):afaf013. doi: 10.1093/ageing/afaf013 (PMC11784590; doi:10.1093/ageing/afaf013)
Supplement: aa-24-1963-File013_afaf013 [file aa-24-1963-file013_afaf013.pdf]

Study title: Behavioural and psychological symptoms of people with dementia in acute hospital settings: a systematic review and meta-analysis

Appendix 8 Summary of treatment outcomes of BPSD in acute hospitals reported in the included studies

Domains of treatment outcomes reported in the included studies	Care procedure	Specialty consultation	Non-pharmacological	Pharmacological management	Falls	Any adverse events	Mortality	Length of stay	Cost	Discharge process	Discharge destination	Emergency admission	Readmission	Carer strains
Aminoff, B. Z. (2016)	O						O							
Berish, D. (2024)													O	O
Eriksson, S. (2007)					O									
Ferreira, A. R. (2022)							O	O					O	
Gilmore-Bykovskyi, A. L. (2021)										O				
Hessler, J. (2018)		O				O								O
Kunik, M. E. (1999)	O													
Nourhashemi, F. (2001)				O								O		
Pitkala, K. H. (2004)	O			O										
Rabins, P. V. (1991)											O			
Sampson, E. L. (2014)						O	O	O	O		O			O
Sommerlad, A (2019)												O		
Spears, C. (2019)		O		O				O						
Tan, L. L. (2005)														O
Tannenbaum, R. (2022)	O	O			O		O	O			O		O	
White, N. (2017)			O	O			O							

Study title: Behavioural and psychological symptoms of people with dementia in acute hospital settings: a systematic review and meta-analysis

More detailed summary of treatments and outcomes associated with BPSD in acute hospital setting

Domains	Treatments and outcomes of BPSD
Care procedure	Compared to people without behavioural symptoms, people with behavioural symptoms were more likely to indwelling bladder catheters (11.1% vs. 6.0%, p < .001), dietary restrictions (41.9% vs. 33.8%, p < .001), bed alarms (81.6% vs. 77.4%, p < .001), chair alarms (34.3% vs. 25.0%, p < .001), DNR orders (40.6% vs. 33.1%, p < .001), ICU stays (14.7% vs. 8.2%, p < .001), and invasive mechanical ventilation (6.5% vs. 2.5%, p < .001). Of those with behavioural symptoms, 73.3% (n = 2645) received an intervention that included restraints, psychotropic medications, or constant observation. (Tannenbaum, R. (2022))<br>Wandering behaviour (35.5% v.s. 18.3%, p=0.02), apathy (51.6% v.s. 21.9%, p<0.001) and insomnia (80.6% v.s. 25.4%, p<0.001) were significantly more common among patients who had been tied with restraints. (Pitkala, K. H. (2004))<br>Depressive symptoms were associated with constant requests for help, complaining and negativism (p<0.001). (Kunik, M. E. (1999))<br>People with 'Not calm' had undergone more invasive procedures (82.9% vs 61.4%, P=0.001); were treated with more antipsychotic drugs (40.2% vs 25.7%, P=.037), antibiotics (70.7% vs 49.5%, P=.004), and infusions (82.9% vs 58.4%, P=.0001). (Aminoff, B. Z. (2016))
Specialty consultation	More likely to have consultation by palliative care (24.8% vs. 17.0%, p < .001), psychiatry (22.4% vs. 5.2%, p < .001), and neurology (19.5% vs. 9.5%, p < .001). (Tannenbaum, R. (2022))<br>Neurology consultation 44 (28%), Psychiatry consultation 14 (9%), Neurology or psychiatry consultation 50 (32%). (Spears, C. (2019))<br>Psychotic symptoms were associated with complicated basic care, increased utilisation of psychiatric or neurologic consults and antipsychotic medication. (Hessler, J. (2018))
Non-pharmacological management	Non-pharmacological management was used in 55% of participants, most commonly psychosocial interventions (36%) with little evidence of monitoring their effectiveness. (White, N. (2017))
Pharmacological management	Among the current medications taken by these patients (n=118), psychotropic drugs were prescribed most commonly (71% of patients), comprising anxiolytics (39%), neuroleptics (25%), hypnotics (18%), and antidepressants (17%). (Nourhashemi, F. (2001))<br>Psychotic symptoms (59.3% vs 37.5%, p<0.001), aggression (25.9% vs 15.0%, p=0.03), wandering behaviour (25.9% vs 14.2%, p=0.02) and insomnia (41.5% vs 21.7%, p<0.001) were significantly more common among patients with antipsychotics and/or sedatives than those without. (Pitkala, K. H. (2004))<br>Antipsychotic medications in 38% of people with dementia, usually quetiapine (25% of admissions), followed by risperidone (7%), haloperidol (7%), and clozapine (2%). Antipsychotics - continued unchanged from home prescriptions in 35 hospitalizations (20%), started in 25 (14%), added/increased in 8 (5%), and

Study title: Behavioural and psychological symptoms of people with dementia in acute hospital settings: a systematic review and meta-analysis

	reduced or omitted in 11 (6%). Benzodiazepines were administered during 50 hospitalizations (28%), most commonly lorazepam (31, 17%). (Spears, C. (2019)) Patients with any form of BPSD during their admission were five times more likely to have an antipsychotic prescribed during the admission (OR 4.99, 95% CI 1.15, 21.70, p=0.032). Antipsychotics were prescribed for 28 (12.2%, 95%CI=8–17) patients, and 19/28 (67.9%) of these prescriptions were new on admission. Prescription of benzodiazepine was associated with anxiety (OR 2.59, 95% CI 1.15, 5.86, p=0.022). Antipsychotic prescription was seven times more likely in patients with aggressive behaviours (OR 7.70, 95% CI 2.25, 26.31, p=0.001). (White, N. (2017))
Falls	Behavioural problems determined by BEHAVE-AD (IRR=1.05, 95% CI 1.004–1.10) was associated with falls. The subcategories of BEHAVE-AD "diurnal rhythm disturbances" (IRR=2.00, 95% CI 1.15-3.50) and "activity disturbances" (IRR=2.38, 95% CI 1.34-4.26) were associated with falls. (Eriksson, S. (2007)) More likely to have fall risk (46.7% vs. 42.3%, p < .002). (Tannenbaum, R. (2022))
Any adverse events	Adverse events (BEHAVE-AD scale 2.9 (3.6) vs 4.4 (5.0), P=0.045) and mortality (BEHAVE-AD scale 4.6 (4.3) vs 2.9 (3.6), p=0.017) were associated with the total severity of BPSD. Possible association between paranoia and adverse events (OR=2.24 (1.14 to 4.41), p=0.019). (Sampson, E. L. (2014)) As for the Psychotic symptoms, delusions were related to more complications than hallucinations. (Hessler, J. (2018))
Mortality	Association between total score of BEHAVE-AD and mortality (OR=1.11 (1.01 to 1.20), p=0.022). Possible association between activity disturbance with adverse events (OR=1.58 (1.09 to 2.28), p=0.015) and mortality (OR=1.56 (1.09 to 2.23), p=0.015). (Sampson, E. L. (2014)) More likely to die during hospitalization (OR: 1.897, CI 95%: 1.571 to 2.291, p< 0.001). (Tannenbaum, R. (2022)) Shorter survival of people with 'Not calm' (Log Rank [Mantel–Cox] P = 0.002). (Aminoff, B. Z. (2016)) Agitation was significantly associated with lowered risk of in-hospital mortality (aOR = 0.648; 95%CI: 0.600–0.700). (Ferreira, A. R. (2022)) Patients who were prescribed antipsychotics, after adjusting for end-of-life medication, age and dementia severity, were significantly more likely to die (adjusted hazard ratio 5.78, 95% CI 1.57, 21.26, p=0.008). (White, N. (2017))
Length of stay (LOS)	Longer LOS (parameter estimate: 2.10, CI 95%: 1.806 to 2.397, p < 0.001). (Tannenbaum, R. (2022)) No association between the length of admission and mean severity of BPSD during the hospital stay. (Sampson, E. L. (2014)) Hospitalizations involving administration of antipsychotics other than quetiapine or clozapine were longer than hospitalizations involving no antipsychotics or only quetiapine or clozapine use (median 7.5 days [IQR 9] vs. 4 days [IQR 4], p = 0.001). (Spears, C. (2019))

Study title: Behavioural and psychological symptoms of people with dementia in acute hospital settings: a systematic review and meta-analysis

	Dementia-related agitation lasted, in general, one day longer (median 9.00 vs. 8.00 days, p<0.001). Agitation (aOR = 1.385; 95%CI:1.314–1.461) increased the odds of longer hospital stays (>8days). (Ferreira, A. R. (2022))
Cost	The association between total cost of the admission to hospital and mean BPSD score was not significant (average increase in cost for each one-point increase of mean BPSD was £215.45, bootstrap 95% CI −348.09 to 1020.37, P= 0.542). (Sampson, E. L. (2014))
Discharge process	Omission rates in discharge communication= 100% for disinhibition, 95.2% for irritability/lability, 94.2% for anxiety, 85.2% for aberrant motor behaviours, and 84.6% for hallucinations; 40% for depression, 89.8% for 1:1 supervision, 89.1% for fall risk, and 90.9% for use of restraints; 12.9% for antipsychotic use. (Gilmore-Bykovskyi, A. L. (2021))
Discharge destination	Discharge destination of 23% of patients with dementia was altered compared with their preadmission place of residence. (Nourhashemi, F. (2001))<br>Less likely to be discharged home (OR: 0.5880, CI95%: 0.534—0.648, p < .001). (Tannenbaum, R. (2022))<br>Nursing care needs were greater for patients with agitation (x2=8.42, df= 1, p=0.003), delirium (x2=11.2, df=1, p=0.0008) and a discharge diagnosis of Alzheimer's disease (x2=20.15, df= 1, p=0.001). Patients with agitation were more likely to be discharged to a nursing home (x2=15.95, df=1, p<0.001). (Rabins, P. V. (1991))<br>Patients with hallucinations were more likely to be discharged to institutional care (OR=1.92 (1.06 to 3.50), p=0.032). (Sampson, E. L. (2014))
Emergency admission	For emergency admission, behavioural problems as the leading cause of admission in people with dementia (26.3%) vs older people without dementia (1.3%). About one third of patients had already been admitted on an emergency basis in the preceding months for similar reasons as the present admission. (Nourhashemi, F. (2001))<br>Emergency hospitalisation rate within the first year after diagnosis was higher in fully adjusted models for those had depressed mood (IRR= 1.14 (1.02, 1.26), p=0.02), but lower rate for elective hospitalisation (IRR = 0.67 (0.53, 0.85), p=0.001). (Sommerlad, A (2019))
Readmission	More likely to be readmitted to the hospital within 30 days (OR: 1.14, CI 95%: 1.014 to 1.289). (Tannenbaum, R. (2022))<br>Patient depression score was associated with hospitalizations at 2-month follow-up (rho = 0.110, p = .030). (Berish, D. (2024))<br>After adjustment for all covariates in the model, agitation was not associated with the risk of readmission (p=0.202). (Ferreira, A. R. (2022))

Study title: Behavioural and psychological symptoms of people with dementia in acute hospital settings: a systematic review and meta-analysis

Carer strains	At the first assessment, 29% of participants had experienced BPSD that were moderately or severely troubling to staff or other carers, this increased to 43% for the whole admission. (Sampson, E. L. (2014))<br>Total distress score in family carer (n = 58) mean ± SD 15.95 ± 8.11 vs Total distress score in professional carer (n = 27) mean ± SD 9.89 ± 6.52 (p-value 0.001). The family caregivers were significantly more distressed than professional caregivers over the delusion (mean(S.D.) = 3.10 (1.01) vs 2.00 (1.47), p= 0.025), agitation (mean(S.D.) = 3.38 (0.92) vs 2.71 (1.15), 0.012), depression (mean(S.D.) = 2.40 (1.10) vs 1.23 (1.17), 0.002), and aberrant motor domains (mean(S.D.) = 2.95 (0.79) vs 2.00 (0.93), 0.009). (Tan, L. L. (2005))<br>Care partner strain was positively associated with hospitalizations at discharge (rho = 0.126, p = .009), 2-month follow-up (rho = 0.104, p = .041), and injuries at 6-month follow-up (rho = 0.113, p = .037). (Berish, D. (2024))<br>Expansive symptoms (aggression, irritability, nighttime disturbances, aberrant motor behaviour and disinhibition) were frequent, distressing for nursing staff and associated with many complications. Affective symptoms (apathy, anxiety and depression) were frequent, non-distressing and associated with few complications. Psychotic symptoms (delusions and hallucinations) were infrequent, distressing and associated with some complications. (Hessler, J. (2018))
